# Human rs75776403 polymorphism links differential phenotypic and clinical outcomes to a CLEC18A p.T151M-driven multiomics

**DOI:** 10.1186/s12929-022-00822-1

**Published:** 2022-06-18

**Authors:** Yu-Wen Hsu, Henry Sung-Ching Wong, Wan-Chen Huang, Yi-Hung Yeh, Chwan-Deng Hsiao, Wei-Chiao Chang, Shie-Liang Hsieh

**Affiliations:** 1 The Ph.D. Program for Translational Medicine, College of Medical Science and Technology, Taipei Medical University and Academia Sinica, Taipei, Taiwan; 2 Department of Clinical Pharmacy, School of Pharmacy, Taipei Medical University, Taipei, Taiwan; 3 Institute of Cellular and Organismic Biology, Academia Sinica, Taipei, Taiwan; 4 Institute of Medical Device and Imaging, National Taiwan University, Taipei, Taiwan; 5 Institute of Molecular Biology, Academia Sinica, Taipei, Taiwan; 6 Department of Pharmacy, Wan Fang Hospital, Taipei Medical University, Taipei, Taiwan; 7 Integrative Research Center in Critical Care, Wan Fang Hospital, Taipei Medical University, Taipei, Taiwan; 8 Genomics Research Center, Academia Sinica, Taipei, Taiwan; 9 Institute of Clinical Medicine, National Yang Ming Chiao Tung University School of Medicine, Taipei, Taiwan; 10 Department of Medical Research and Education, Taipei Veterans General Hospital, Taipei, Taiwan; 11 Graduate of Institute of Cancer Biology and Drug Discovery, Taipei Medical University, Taipei, Taiwan

**Keywords:** CLEC18A, rs75776403, CLEC18A p.T151M, Phosphatidic acid (PA), Phosphatidylserine (PS), Thyroid hormone, Body height

## Abstract

**Background:**

Human traits, diseases susceptibility, and clinical outcomes vary hugely among individuals. Despite a fundamental understanding of genetic (or environmental) contributions, the detailed mechanisms of how genetic variation impacts molecular or cellular behaviours of a gene, and subsequently leads to such variability remain poorly understood.

**Methods:**

Here, in addition to phenome-wide correlations, we leveraged multiomics to exploit mechanistic links, from genetic polymorphism to protein structural or functional changes and a cross-omics perturbation landscape of a germline variant.

**Results:**

We identified a missense *cis*-acting expression quantitative trait locus in *CLEC18A* (rs75776403) in which the altered residue (T_151_→M_151_) disrupts the lipid-binding ability of the protein domain. The altered allele carriage led to a metabolic and proliferative shift, as well as immune deactivation, therefore determines human anthropometrics (body height), kidney, and hematological traits.

**Conclusions:**

Collectively, we uncovered genetic pleiotropy in human complex traits and diseases via *CLEC18A* rs75776403-regulated pathways.

**Supplementary Information:**

The online version contains supplementary material available at 10.1186/s12929-022-00822-1.

## Background

Insights acquired from genetic association studies have greatly advanced our understanding of the biology of human traits and the pathophysiology of numerous complex diseases. Combining those studies with pathway analyses has further shed light on the underlying mechanisms that drive traits and/or disease variability among individuals. Due to the statistical limitation of such genetic epidemiological methodologies, it is still a big challenge to parse the molecular and cellular processes that link obscure genetic variation to explicit phenotypic changes. Variability in gene expression imposed by germline variation has been revealed by the identification of *cis*-acting expression quantitative trait locus (*cis*-eQTL). Moreover, gene set enrichment analysis permits a multigene (horizontal) view of the occurrence of human traits. Despite this, a full and integrative links (multiomics or vertical view) from genetic changes to subcellular molecular features (transcriptional or translational products, phosphorylation sites, metabolites, and biological pathways), and ultimately to phenotypic changes remains a formidable task.

C-type lectin 18 (CLEC18) family comprise *CLEC18A* (16q22.1), *CLEC18C* (16q22.1), and *CLEC18B* (16q22.3). Each of CLEC18 family member encodes a 446 amino acid polypeptide comprising a C-terminal carbohydrate recognition domain (CRD), two epidermal growth factor (EGF) and (EGF)-like domains, and a N-terminal cysteine-rich secretory protein/antigen 5/pathogenesis related-1 (CAP) domain, also known as Sperm-coating protein (SCP) or Tpx antigen 5/pathogenesis related-1/Sc7 (TAPS) domain. CLEC18 protein has been shown to localized in intracellular organelles such as Golgi apparatus, endoplasmic reticulum (ER), and early endosomes, and can also be found in the extracellular milieu [1]. Recent studies revealed the implications of the involvement of CLEC18 in host defence against H5N1 (especially for *CLEC18A*) [2], hepatitis B virus (HBV) [3] and hepatitis C virus [4] infections. Despite this, an exhaustive characterization of the pathophysiological roles of CLEC18 genes is still lacking.

Inferring the functions of CLEC18 genes can be achieved via inspecting the polymorphic amino acid in protein domains, and studying the influence of genetic variant on protein structure and functional change. For examples, the polymorphic residues at the CRD domain of CLEC18A (S339) and CLEC18A-1 (R339), and the p.405LVWLSAAMG insertion in CLEC18B CRD domain resulted in the loss of glycan-binding affinity [1]. In this study, we aimed to further identify crucial genetic polymorphism(s) among the CLEC18 gene family in humans. In addition to in silico prediction and domain structural modelling, we further validate the impacts of variants on CLEC18 genes by lipid-binding assay. Moreover, variant-associated profiles and the enriched pathways of multiomics, including phenomes, transcriptomes, proteomes, metabolomes, and phosphoproteomes, were further characterized. This approach provides a framework to illustrate the mechanistic (molecular and/or cellular) details of a genetic polymorphism, and reveals the power of integrating multiple-omics in a genetic epidemiological study to improve our understanding on the impact of genetic variant in human traits and diseases.

## Materials and methods

### Prioritizing the CLEC18A p.T151M residue and functional predictions

CLEC18 family genes (*CLEC18A*, *CLEC18B*, and *CLEC18C*) genotype data from 2504 samples were queried from the 1000 Genome (1000G) Phase 3 database (https://www.internationalgenome.org/; accessed Dec 2, 2016). These 1000G individuals were categorized into five major populations of African, Admixed American, European, East Asian, and South Asian.

#### Missense annotation

Information (missense or not) regarding CLEC18 family gene variants was obtained from Haploreg v4.1.

#### Expression quantitative trait locus (*cis*-eQTL) annotation

Tissue-specific *cis*-eQTLs of the *CLEC18A*, *CLEC18B*, and *CLEC18C* genes were queried from the Genotype-Tissue Expression (GTEx) Portal v8 (http://www.gtexportal.org/home/; accessed Aug 27, 2019).

#### Prediction of deleteriousness

Computational predictions of the impacts of missense variants were built based on the biochemical properties of amino acid substitutions. To evaluate the deleteriousness to protein function and the structure of amino acid substitutions, we adopted three in silico prediction tools including Sort Intolerant From Tolerant (SIFT; https://sift.bii.a-star.edu.sg/; accessed Oct 2019) [5], Polymorphism Phenotyping v2 (PolyPhen-2; http://genetics.bwh.harvard.edu/pph2/; accessed Oct 2019) [6], and Combined Annotation Dependent Depletion (CADD; https://cadd.gs.washington.edu/snv/; accessed Aug 2021) [7, 8]. The SIFT score ranges 0 ~ 1. The cutoff score for SIFT is 0.05. A substitution is predicted to be tolerated (or deleterious) with a SIFT score of > 0.05 (or < 0.05). PolyPhen-2 uses the same score range as SIFT, but in the opposite direction, with a score closer to 1 representing high confidence of being damaging. CADD computes scores for all potential genetic variants throughout the reference genome. The raw score is further transformed into a CADD Phred score by ranking the variants for all 8.6 billion genetic variants. CADD Phred scores range 1 ~ 99. Phred scores for ranking the top 10% of causal genetic variants are assigned as 10. The top 1% are assigned as 20, etc. With a reasonable cutoff for deleteriousness ranging 10 ~ 20, we arbitrarily defined variants with a Phred score of > 20 as deleterious and < 10 as non-deleterious.

The *CLEC18A* gene plot was directly adopted from the Biodalliance website (http://www.biodalliance.org/index.html; accessed Feb 7, 2021).

### Structural homology modelling of the CLEC18A protein domain

We constructed a three-dimensional (3D) homology model by inputting the CAP/SCP/TAPS domain sequences of CLEC18A via the SWISS MODEL server [9]. Briefly, the algorithm identifies a “template protein”, a sequence homologue of the input sequence, and uses the template protein to build a 3D model. In this case, human glioma pathogenesis-related protein 1 (GLIPR1) was selected as the template protein for model construction and further structural analysis. Visual rendering of 3D homology models was performed using PyMol v2.4.2 (https://pymol.org/2/; accessed Oct 26, 2021).

### Lipid-binding affinity test for wild-type and mutant form CLEC18A residue

DNA fragments of the CAP/SCP/TAPS domain, which encoded the wild-type (WT; p.T151) and mutant form (p.M151) of the *CLEC18A* residue, were amplified by a reverse-transcription polymerase chain reaction (RT-PCR) and subcloned into the pcDNA3.1-hIgG1 Fc (mut) vector to generate the WT and mutant CLEC18A.Fc fusion proteins. The FreeStyle 293 Expression System (Invitrogen, Carlsbad, CA, USA) was applied to overexpress the CLEC18A.Fc fusion proteins. The detailed procedures were described in our previous study [1].

To perform the protein-lipid overlay assay, the phosphorylated derivatives of phosphatidylinositol (PIP) strips (P23751) membranes and Sphingo strips (S23753) membranes were purchased from Thermo Fisher Scientific (Waltham, MA, USA). Light exposure of the lipid strip membranes was avoided during the entire process before detection. The lipid strip membranes were initially blocked in 3% bovine serum albumin (BSA) in phosphate-buffered saline (PBS) for 1 h (h) at room temperature. Next, the SCP domain of the WT and mutant CLEC18A.Fc fusion proteins were added at final concentrations of 500 and 50 ng/ml for 2 h of incubation at room temperature. After three washes with 3% BSA-PBST (PBS plus 0.1% Tween20), the lipid membranes were incubated with an anti-human immunoglobulin G (IgG) horseradish peroxidase (HRP) antibody (1:5000) in blocking buffer for 1 h at room temperature. After three washes with 3% BSA-PBST, the lipids were detected with enhanced chemiluminescence reagents (GERPN2235, Cytiva, Chicago, IL, USA).

### Data from the Taiwan biobank (TWB)

The Taiwan Biobank (TWB), a nationwide research database in Taiwan, was launched to facilitate biomedical research and further translate work into clinical settings by incorporating genomic, environmental, and disease profiles [10].

Participants were recruited from local communities, with inclusion criteria of being aged 30 ~ 70 years, physically active, without a cancer history, and self-reported to be of Han ancestry (i.e., both parents). Clinical (and follow up) data from anthropometric measurements, biospecimen tests, physical examinations, and questionnaires were obtained during sample enrollment. Written informed consent was provided by all individuals who participated in the TWB project. Ethical approval was obtained from the Institutional Review Board (IRB) of Taipei Medical University (IRB no. N201906005), the Ethics and Governance Council of the TWB (TWBR10807-05, TWBR10906-03), and Academia Sinica (AS-IRB01-16,018).

### CLEC18A p.T151M genotypic data of the TWB cohort

Genomic DNA of participants in the TWB project was harvested using a standardized protocol and subjected to genotyping using the Axiom Genome-Wide TWB v2.0 Array Plate. Stringent quality-control filters for TWB biallelic SNV and indel genotype data were applied using PLINK v1.9 [11]. Variants with heterozygous haploid genotypes were addressed by (i) checking heterozygous calls in the pseudoautosomal region of chromosome (chr) X in males; (ii) sex checking; and (iii) directly removing the variants. Subjects with ambiguous sex data were removed during sex checks. Individuals and genotypes were sequentially filtered with a call rate threshold of 98%. Variants with a minor allele frequency (MAF) of < 1% and a Hardy–Weinberg equilibrium (HWE) *P* value of < 10^–10^ were further discarded.

Next, a subset of independent variants was selected through linkage disequilibrium (LD) pruning by calculating the pairwise LD of autosomal variants (MAF > 10%) with parameters of a window size of 200 kb, a step size of 5, and a variance inflation factor threshold of 0.2. Outliers were detected and filtered according to the heterozygosity rate calculated from the pruned variant subset. Genetic relationships among individuals were estimated using Genome-wide Complex Trait Analysis (GCTA) [12], followed by calculating the principal components (PCs).

Local imputation of rs75776403 (chr16:69954569; GRCh38) was performed by first extracting biallelic single-nucleotide polymorphisms (SNPs) of around ± 2.5 Mb. Next, phasing and imputation were conducted using the Michigan Imputation Server (https://imputationserver.sph.umich.edu/index.html#!; accessed Dec 2, 2021) by choosing the 1000G Phase 3 (v5) East Asian population as the reference. Post-imputation quality filtering was applied with an imputation score of > 0.8 and MAF of > 5%.

### Phenotypic (clinical) profiles of the TWB cohort

Phenotypic varieties of TWB participants were collected from test data of biological specimens (blood and urine), physical examinations, and questionnaires. The phenotype data were further processed as follows.

#### Quantitative traits

In total, 86 quantitative traits were included in our study: (i) 62 original traits (spanning 11 phenotype categories) available from clinical profiles of the TWB cohorts, including five anthropometric (height [abbreviated as Ht], weight [Wt], body fat [BF], waist [WC] and hip [HC] circumference), three cardiac (systolic [Sys] and diastolic [Dias] blood pressure, heartbeat speed [Heartbeat]), two gynecological (age of menopause [Menps], age of menarche [Menrc]), three habitual (alcohol intake [DRK_y], nut intake [Nut_DsYr], smoke intake [SMK_PkYr]), five hematological (red blood cell count [RBC], white blood cell count [WBC], platelet count [Plt], hemoglobin [Hb], hematocrit [Hct]), six hepatic (total bilirubin [TBil], serum albumin [Alb], aspartate aminotransferase [AST], alanine aminotransferase [ALT], gamma-glutamyl transferase [GGT], alpha fetoprotein [AFP]), six metabolic (glycated hemoglobin [HbA1c], fasting glucose [FGlu], total cholesterol [TCho], triglyceride [TG], high- [HDLc] and low-density [LDLc] lipoprotein cholesterol), four nephrotic (blood urea nitrogen [BUN], creatinine [Cr], uric acid [UA], microalbumin [mAlb]), five orthopedic (stiffness index [SI], T score [T], Z score [Z], speed of sound [SOS], broadband ultrasound attenuation [BUA]), 18 pulmonary (vital capacity [VC], tidal volume [TV], expiratory reserve volume [ERV], inspiratory reserve volume [IRV], inspiratory capacity [IC], VC-to-Ht ratio [VcHtRatio], forced VC [FVC], forced EV in 1 s [FEV1], FEV1-to-FVC ratio [Fev1FvcRatio], FEV1-to-VC ratio [Fev1VcRatio], mean maximal flow [MMF], peak expiratory flow [PEF], 25% forced expiratory flow [FEF25], 50% FEF [FEF50], 75% FEF [FEF75], FEF75-to-Ht ratio [Fef75HtRatio], extrapolated volume-to-FVC ratio [EvFvcRatio], forced inspiratory volume in 1 s-to-FVC ratio [Fiv1FvcRatio]), and 5 virological (hepatitis C virus antibody [AtHCVAb], hepatitis B surface antigen [HBsAg], hepatitis B e antigen [HBeAg], hepatitis B surface antibody [AtHBsAb], hepatitis B core antibody [AtHBcAb]).

(ii) Additional 24 traits were derived from original traits, including nine anthropometric (body-mass index [BMI], corpulence index [CI], waist-to-stature ratio [WSR], waist-to-hip ratio [WHR], body adiposity index [BAI], BMI-adjusted WHR [BMIAdjWHR], BMI-adjusted waist circumference [BMIAdjWC], BMI-adjusted hip circumference [BMIAdjHC], height-adjusted BMI [HtAdjBMI]), three cardiac (pulse pressure [Pulse], systolic-to-diastolic blood pressure ratio [SysDiasRatio], diastolic-to-pulse pressure ratio [DiasPulseRatio]), one hematological (hematocrit-to-hemoglobin ratio [HctHbRatio]), four metabolic (BMI-adjusted fasting glucose [BMIAdjFGlu], fasting glucose-to-glycated hemoglobin ratio [FGluHbA1cRatio], triglycerides-to-high density lipoprotein cholesterol ratio [TgHDLcRatio], total cholesterol-to-high density lipoprotein cholesterol ratio [TChoHDLcRatio]), three hepatic (aspartate aminotransferase-to-alanine aminotransferase ratio [AstAltRatio], gamma-glutamyl transpeptidase-to-alanine aminotransferase ratio [GgtAltRatio], alpha fetoprotein-to-transaminase ratio [AfpAstAltRatio]), and four nephrotic parameters (glomerular filtration rate based on serum creatinine (estimated using four-variable Modification of Diet in Renal Disease (MDRD) study equation and further cropped into 15 ~ 200) [eGFR], uric acid-to-creatinine ratio [UaCrRatio], blood urea nitrogen-to-creatinine ratio [BunCrRatio] and microalbumin-to-creatinine ratio [mAlbCrRatio]).

Notably, for sitting systolic/diastolic pressures and heartbeat speed, values were averaged across three measurement time points. If a quantitative trait presented a censored value due to the detection limit, we directly replaced the record with the censored value.

#### Binary traits

In total, 84 binary traits were included: three alimentary canal diseases (gastroesophageal reflux [GasRef], irritable bowel syndrome [IBS], peptic ulcer [PepUlc]), seven arthritis (arthritis [Arthr], adhesive capsulitis [AC], ankylosing spondylitis [AS], degenerative arthritis [DA], palindromic rheumatism [PR], psoriatic arthritis [PsA], rheumatoid arthritis [RA]), one bone disease (osteoporosis [Osteo]), 11 cancers (breast cancer [Brst], cervical cancer [Cerv], colorectal cancer [Colon], gastric cancer [Gast],liver cancer [Liv], lung cancer [Lung], nasopharyngeal cancer [Nasph], ovarian cancer [Ova], uterine cancer [Uter], prostate cancer [Prst], other cancer [OtherCA]), six cardiovascular diseases (arrhythmia [Arythm], congenital heart disease [CongHrt], coronary artery disease [CoroArt], cardiomyopathy [Crdmyo], valve heart disease [ValHrt], other heart disease [OtherHrt]), seven gynecologic (dysmenorrhea [Dys], endometriosis [Endo], ovarian cyst [OvaCys], hormone drug usage for menopause [HorDrgMenps], irregular menstruation [IrgMS], myoma [Myo], natural abortion [NatAbor]), seven habitual (coffee consumption [Cof], tea consumption [Tea], snake consumption [Snak], drink [DRK], nut [NUT], smoke [SMK], sport [Spt]), three headache-induced symptoms (affect diary life when headache [AchDiary], nausea when headache [AchNau], photophobia when headache [AchePhot]), one hepatic symptom (liver gall stone [LivGaStn]), one immune disease (type 1 diabetes mellitus [tp1DM]), seven metabolism disorders (diabetes mellitus [DM], type 2 diabetes mellitus [tp2DM], gestational diabetes mellitus [tyGDM], gout [Gout], hyperlipidemia [HypLip], hypertension [HypTens], stroke [Stroke]), two nephrotic symptoms (kidney stone [KdnStn], renal failure [RenlFail]), six nervous-system diseases (dementia [Dmntia], epilepsy [Epilps], hemicrania [Hemcra], multiple sclerosis [MS], Parkinson’s disease [Prkins], vertigo [Vrtig]), eight ophthalmic symptoms (blind [Blnd], color blind [ColBlnd], cataract [Ctrc], floaters [Flt], glaucoma [Gluc], retinal detachment [RntDe], xerophthalmia [Xrph], other eye disease [OtherEye]), six psychiatric disorders (alcoholism/drug abuse [AlcoDrgAbuse], depression [Deprs], manic depression [ManDeprs], obsessive compulsive disease [ObsComp], postpartum depression [PostDeprs], schizophrenia [Schiz]), two pulmonary syndromes (asthma [Asthm], emphysema bronchitis [EmphBrnch]), and six soreness parameters (articulus ache [Art], back waist ache [BakWst], headache [Head], neckache [Nck], sciatica [Sct], other ache [OtherAch]).

#### Ordinal traits

In total, 19 ordered traits were also included: one habitual (snake consumption frequency [SNKFrq]), seven ophthalmologic (cataract eye number [CtrcN], blind eye number [BlndN], color blind eye number [ColBlndN], floaters eye number [FltN], glaucoma eye number [GlucN], retinal detachment eye number [RtDeN], xerophthalmia eye number [XrphN]), seven soreness (headache frequency [HeadFrq], headache severity [HeadSvr], neck-ache frequency [NckFrq], back waist ache frequency [BakWstFrq], articulus ache frequency [ArtFrq], sciatica frequency [SctFrq], dysmenorrhea frequency [DsmFrq]), and four psychiatric condition levels (nervousness [Nerv], feeling down [Dwn], anxiety [Anxt], depression [Dprs]).

### Phenome-wide association study (PheWAS) of rs75776403 in Taiwanese Hans

We conducted a phenome-wide association study (PheWAS) of rs75776403 using the TWB cohort. First, a list of genetically unrelated samples by filtering out 1st- or 2nd-order relationships was obtained using the “–unrelated” option implemented in KING v2.2.7 [13]. Next, association tests were conducted by incorporating covariates of age, squared age, sex (ignored in sex-restricted traits), and the top 20 PCs. The association model was determined according to the type of trait.

#### Quantitative traits

For the 86 quantitative traits, a linear regression model was adopted. The traits were normalized using the inverse normal transformation (Elfving method) before fitting the association model. Hypothesis testing was conducted using the type I (sequential) method.

#### Binary traits

For the 84 dichotomized traits, a logistic regression model was applied.

#### Ordinal traits

For the 19 ordinal traits, an ordered logistic regression model was applied using the “polr” function implemented in the MASS package. The *Z* statistics were calculated using the “coeftest” function implemented in the lmtest package.

To avoid false positivity, a local false discovery rate (FDR) was estimated using the “lfdr” function implemented in the qvalue package.

### Validation of PheWAS results

Summary statistics regarding the associations between rs75776403 and 2173 traits from UK Biobank (UKB, ~ 456 K subjects of European ancestry) were directly adopted from the GCTA website (https://yanglab.westlake.edu.cn/software/gcta/#DataResource; accessed Dec 25, 2021) [14, 15]. Moreover, summary statistics of rs75776403 associations with 229 traits in a Japanese population were adopted from Biobank Japan (BBJ, ~ 178 K individuals) and pheweb.jp website (https://pheweb.jp/downloads; accessed Dec 27, 2021) [16].

#### Meta-analysis

A fixed-effect or random-effect model was applied for a trans-ethnic meta-analysis across different populations using a restricted maximum likelihood to estimate the heterogeneity variance. Knapp-Hartung adjustments were further applied for the random-effect model. Statistical tests and forest plot visualization were conducted using the meta package.

### Cancer cell line encyclopedia (CCLE) multiomics

We queried multiple omics data (including genomic, transcriptomic, proteomic, metabolomic, and phosphoproteomic profiles) of the CCLE from the Dependency Map (DepMap) portal (https://depmap.org/portal/; accessed Oct 28, 2021) [17]. The data preprocessing and analytical steps of each profile are described as follows.

#### Genomic data

Raw genomic data (Affymetrix Genome-Wide Human SNP 6.0 Array) from CCLE samples in Affymetrix CEL format were converted to Affymetrix CHP and then binary variant call format using the affy2vcf (https://github.com/freeseek/gtc2vcf) tool. Variants (SNPs and short insertion/deletions (indels)) were normalized according to the human genome reference assembly 38 (GRCh38). We next extracted only biallelic SNPs that satisfied the following criteria: (i) autosomal or chrX; (ii) call rate of > 98%; (iii) MAF of > 1%; (iv) non-somatic (according to CCLE mutations (~ 1.3 M somatic calls across 1741 cell lines) from whole-exome sequencing profiles); and (v) showing LD of *r*^*2*^ > 0.4 to at least one of the nearby 50 SNPs (within 1000 Kb). After excluding cell lines that failed the heterogeneity test, these putative “germline” variants were next subjected to genotype imputation through the Michigan Imputation Server by using 1000G Phase 3 v5 as a reference. Finally, variants with a post-imputation info score of > 0.3 and a MAF of > 5% were selected for association analyses.

#### Transcriptomic and proteomic data

For normalized CCLE transcriptomic (mRNA sequencing) and proteomic (quantitative multiplexed proteomics profiles by mass spectrometry from the Gygi lab (https://gygi.hms.harvard.edu/index.html; accessed Dec 14, 2021) [18] data, gene symbols were first harmonized using the HGNChelper package. Next, the expression values of duplicate genes were mean-aggregated.

#### Metabolomic data

Data regarding CCLE metabolomes containing expression values for 228 metabolites [19] were directly downloaded from the DepMap website.

#### Phosphoproteomic data

Data on phosphorylation sites (p-sites) across ~ 10 K genes (by trypsin and/or GluC digestion) were directly adopted from M. Frejno et al*.* [20]. Gene names were harmonized using the HGNChelper package.

Herein, we adopted the general term “molecular feature” to indicate mRNA, protein, metabolite, and p-site of multiomics. Associations between rs75776403 and molecular features of each omic profile were conducted as follows: (i) molecular features with zero variance were removed; (ii) a linear model between a molecular feature (as a dependent variable) and rs75776403 (as the independent variable) was fitted by including sex, age, histology, ethnicity, pathology and primary cancer type as covariates, with the type I (sequential) significance was assessed using an exact permutation test with the “lmp” function implemented in the lmPerm package; and (iii) a differentially expressed molecular feature was defined based on a significant threshold of *P* = 0.01.

### Gene set and pathway enrichment analysis

#### Transcriptome and proteome

We conducted gene set enrichment analysis (GSEA) to test for the enrichment of pathways (and/or gene sets) in expression data. A fast algorithm (*i.e.* Fast Gene Set Enrichment Analysis; FGSEA) implemented in the fgsea package was adopted with using weighted differential expression test statistics (*i.e.* − log_10_(*P*) × *β*; where *P* is the significant value and *β* is the regression coefficient of a molecular feature) from differentially expressed test results as input. Pathways (and/or gene sets) used for the test were compiled from the Kyoto Encyclopedia of Genes and Genomes (KEGG) database, gene ontology (GO) biological process (BP) database, and Reactome pathway database. Significant enrichment was defined as *P* ≤ 0.01 and the direction of enrichment was confirmed using a normalized enrichment score (NES of > 0 for positively enriched and < 0 for negatively enriched).

Robust expression enrichment was defined as follows: the weighted differential expression test statistics of each omic were first scaled. Next, pathways with scaled statistics of > 2 or < (− 2) in both expression omics were considered to be robustly enriched.

#### Phosphoproteome

The weighted differential expression test statistics of p-sites from trypsin and gluC were separately calculated, and then aggregated by the maximum, which takes account of the difference in site-specificity of the two digestive enzymes. The weighted statistics of each digestive enzyme and the merged weighted statistics were subjected to GSEA analysis. Reference pathways regarding human phosphosite-specific signatures were adopted from PTMsigDB v1.9.0 (https://github.com/broadinstitute/ssGSEA2.0; accessed 30 Dec 2021) [21]. After harmonizing gene symbols using the HGNChelper package, we conducted the FGSEA as described above.

### Tissue-specific mRNA expression data

The mRNA-sequencing (Seq) expression profiles in transcript per million of several tissues, including the adrenal gland, brain, liver, ovary, pituitary, testis, and thyroid were queried from the Genotype-Tissue Expression (GTEx; v8) portal (https://gtexportal.org/home/datasets; accessed Nov 29, 2021). Gene symbols were harmonized using the HGNChelper package. Next, expression values of duplicated genes were aggregated by the maximum. A Spearman's rho (*ρ*) statistic was calculated to assess the rank-based association of pairwise genes.

## Results

### Prioritization of p.T151M (rs75776403) as a deleterious variant in CLEC18A

**Fig. 1:**
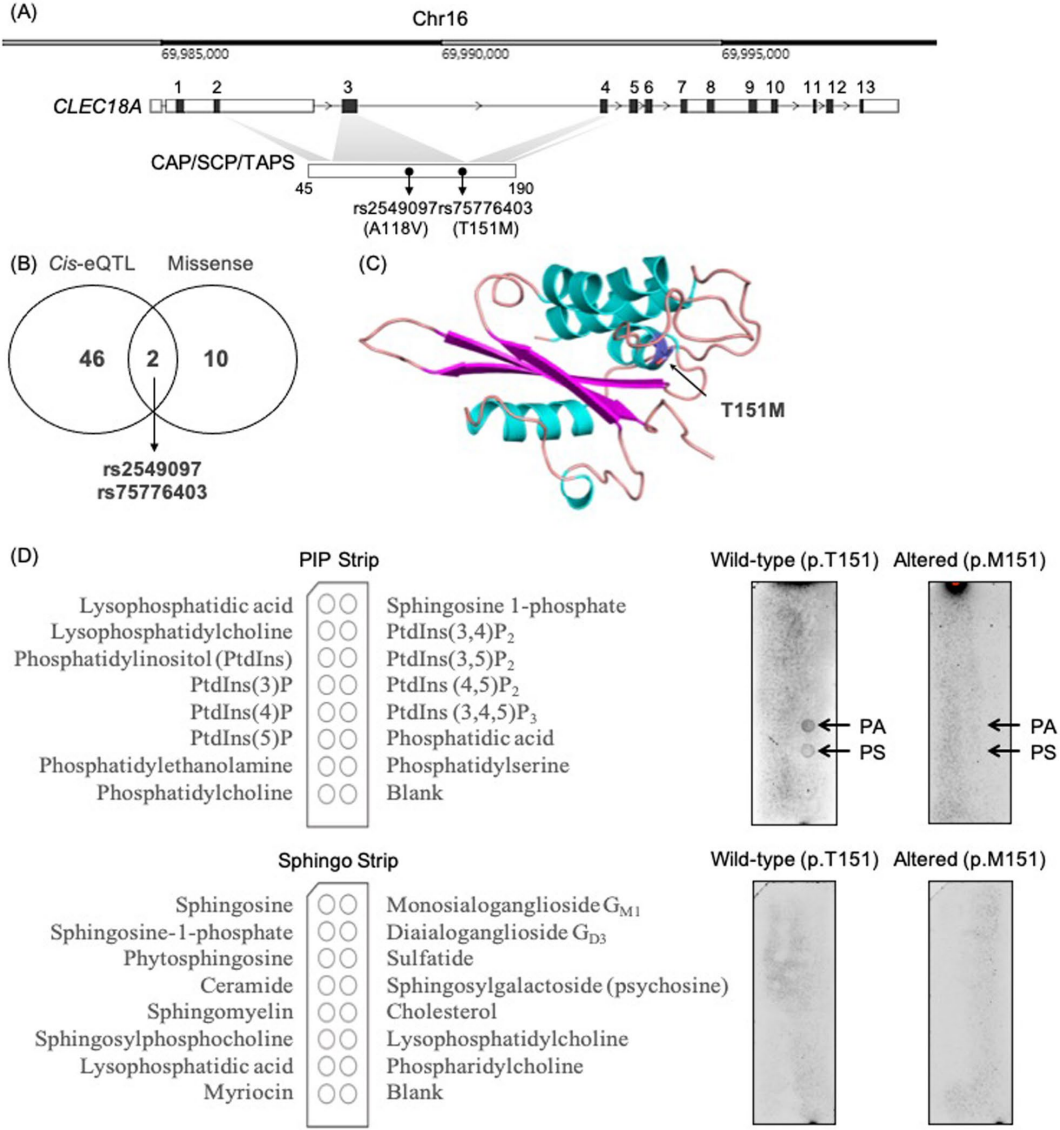
The *CLEC18A* rs75776403 (p.T151M) polymorphism. **A** Genetic view and rs2549097 or rs75776403 variants of the human *CLEC18A* gene. **B** Venn diagram showing overlapping of *cis*-eQTLs and missense variants of CLEC18 family genes. **C** The 3D homology model of the CLEC18A CAP/SCP/TAPS protein domain. The residue 151 of the CLEC18A CAP/SCP/TAPS protein domain is indicated by an arrow. **D** Protein-lipid overlay assay for wild-type (p.T151; left) and altered (p.M151; right) CLEC18A protein

To identify the genetic variants that impact CLEC18 family genes functionally, we screened SNPs across the *CLEC18A*, *CLEC18C*, and *CLEC18B* genes based on the three criteria: (1) common (minor allele frequency (MAF) > 1%) in at least one ethnic population; (2) missense (changing amino acid residue of the gene; Additional file 1: Table S1); and (3) *cis*-regulatory (*a.k.a.* expression quantitative trait locus (*cis*-eQTLs); Additional file 1: Table S2). Of 48 *cis*-eQTLs that were associated with the primordial gene expression levels of *CLEC18A/B/C* genes as annotated by the Genotype-Tissue Expression (GTEx) database, two SNPs (*i.e.*, rs2549097 (p.A118V) and rs75776403 (p.T151M)) on *CLEC18A* were found to be missense, and thus were considered to be putative variants with functional impacts (Figs. 1A-B). Regarding to the *CLEC18B* and *CLEC18C*, we did not identify any putative functional variants.

As shown in the Fig. 1A, the rs2549097 (chr16:69,954,470; GRCh38) c.C-to-T allelic change corresponded to p.A118V (alanine-to-valine at position 118), and the rs75776403 (chr16:69954569; GRCh38) c.C-to-T corresponded to p.T151M (threonine-to-methionine at position 151). To parse functional impacts of the SNPs, in silico predictions were conducted to prioritize rs75776403 (but not rs2549097) as deleterious for *CLEC18A* (Table 1). In detail, the deleteriousness of rs2549097 and rs75776403 was predicted using the Combined Annotation Dependent Depletion (CADD), Sorting Intolerant From Tolerant (SIFT) and Polymorphism Phenotyping v2 (PolyPhen-2) tools. Accordingly, rs2549097 was predicted to be tolerated by the protein function and/or structure (CADD Phred score = 0.245 [non-deleterious]; SIFT score = 0.06 [tolerated]; PolyPhen-2 score = 0 [benign]), whereas rs75776403 was predicted to be damaging (CADD Phred score = 22.7 [deleterious]; SIFT score = 0 [deleterious]; PolyPhen-2 score = 0.998 [probable damage]). Therefore, we further focused on rs75776403 (*CLEC18A* p.T151M), a *cis*-eQTL in four (testis, artery, brain, and adrenal gland) tissues (Additional file 1: Table S3 and Fig. S1), for the downstream analysis. It is noteworthy that the terms “rs75776403 c.C-to-T” (DNA-level), “*CLEC18A* c.452C-to-T” (DNA-level), “rs75776403 p.T151M” (protein-level) and “*CLEC18A* p.T151M” (protein-level) were considered interchangeable.

**Table 1:** Functional prediction of rs2549097 and rs75776403 in *CLEC18A*

Variant	rs2549097	rs75776403
Allelic change	c.353C > T [GCG → GTG]	c.452C > T [ACG → ATG]
Amino acid change	p.A118V	p.T151M
Protein domain	CAP/SCP/TAPS	CAP/SCP/TAPS
SIFT	0.06 [Tolerated]	0 [Deleterious]
PolyPhen-2	0.000 [Benign]	0.998 [Probably damaging]
CADD Phred	0.245 [Non-deleterious]	22.7 [Deleterious]

*CAP* C-terminal cysteine-rich secretory protein/antigen 5/pathogenesis related-1, *SCP* Sperm-coating protein, *TAPS* Tpx antigen 5/pathogenesis related-1/Sc7, *SIFT* Sort Intolerant From Tolerant, *PolyPhen-2* Polymorphism Phenotyping v2, *CADD* Combined Annotation Dependent Depletion

### CLEC18A p.T151M disrupted the lipid-binding affinity of the CAP/SCP/TAPS domain

The rs75776403 residue (p.M151 was considered as a “mutant” form compared to “wild-type” p.T151) was located on the C-terminal cysteine-rich secretory protein/antigen 5/pathogenesis related-1 (CAP) or Sperm-coating protein (SCP) or Tpx antigen 5/pathogenesis related-1/Sc7 (TAPS) domain of CLEC18A. To assess the possible location of T151 of CLEC18A, we have to establish the protein structure. However, the precise tertiary structures of the SCP domain of CLEC18A remain unknown, we, therefore, generated a homologous model via the SWISS-MODEL Server [9]. From the homology modelling results, residue T151 of CLEC18A is located on the outer surface of the CAP/SCP/TAPS domain. Furthermore, this residue is located in the bottom cavity of the folded protein domain. Taken together, these results illustrated the accessibility of the residue to other interacting substrates of CLEC18A (Fig. 1C). Since threonine-to-methionine (polar uncharged to neutral hydrophobic) conversion may abrogate the polarity of the residue 151 of CLEC18A, we speculated that rs75776403 (p.T151M) disrupted the substrate-binding affinity of the CLEC18A protein domain. Given that the sterol- and/or acidic glycolipid-binding (and exportation) function was conserved across SCP/TAPS/CAP proteins [1, 22], we next tested whether p.T151M might disrupt the lipid-binding ability of the CAP/SCP/TAPS domain of CLEC18A (Fig. 1D). As a result, WT CLEC18A showed binding affinity to two acidic phospholipids, *i.e.*, phosphatidic acid (PA) and phosphatidylserine (PS). Specifically, the ability to bind PA and PS was abolished in CLEC18A with p.M151, suggesting functional disruption by the missense rs75776403 in the CAP/SCP/TAPS domain.

### Phenotypic landscape of rs75776403 in different human populations

Given the structural and functional perturbation of rs75776403 (p.T151M) in the CAP/SCP/TAPS domain of CLEC18A, we next clarified the genetic epidemiological links in humans. First, the allelic frequencies of rs75776403 in different ethnicities were summarized: rs75776403 c.T allele (corresponding to p.M151) was the most abundant in Asians (48%), followed by admixed Americans (32%), Europeans (27%) and Africans (11%). Specifically, the c.T allelic frequency of rs75776403 was 47% in Taiwanese Hans.

Second, we conducted a phenome-wide association study (PheWAS) of rs75776403 in the Taiwanese population to examine the correlations between rs75776403 and multifarious human traits. By leveraging a Taiwan Biobank (TWB) cohort with a sample size of 68,080, we parsed the genetic association profile of rs75776403 in 189 traits as follows: (i) Among quantitative traits (Additional file 1: Table S4), 62 traits were directly adopted from clinical data (compiled from blood or urine specimens, physical examinations, and questionnaires) of TWB subjects. Next, 24 traits with clinical relevance were derived and further included (Additional file 1: Table S5), resulting in a total number of 86 quantitative traits spanning 11 phenotype categories (Additional file 1: Table S6). Similarly, (ii) 84 dichotomized (binary; Additional file 1: Table S7-S8) and (iii) 19 ordinal (Additional file 1: Table S9-S10) traits were also included in the study. Consequently, we detected significant statistical correlations of rs75776403 c.C-to-T (corresponding to *CLEC18A* p.T151-to-M151) with quantitative traits including three anthropometric (smaller body height [Ht], local false discovery rate (Fdr) = 4.52 × 10^–4^; lower body weight [Wt], Fdr = 8.64 × 10^–3^; and increase waist-to-stature ratio [WSR], Fdr = 3.34 × 10^–2^), three nephrotic (decreased creatinine [Cr], Fdr = 6.16 × 10^–5^; decreased blood urea nitrogen [BUN], Fdr = 7.18 × 10^–4^; and elevated eGFR, Fdr = 1.15 × 10^–3^), one hematological (elevated platelet count [Plt], Fdr = 3.22 × 10^–4^) and one hepatic parameter (*α*-fetoprotein-to-transaminase ratio [AfpAstAltRatio], Fdr = 2.68 × 10^–2^). In addition, traits such as the risk for cardiomyopathy [Crdmyo] (Fdr = 1.80 × 10^–2^), and retinal detachment eye number [RtDeN] (Fdr = 6.11 × 10^–6^) were also significantly associated with the rs75776403 c.C-to-T polymorphism (Table 2).

**Table 2:** Phenome-wide association study for *CLEC18A* rs75776403 in Taiwanese Hans from the Taiwan Biobank (TWB)

Category	Trait	Type	Sample No	*β*	Std. Err	Fdr
Anthropometric	Ht	Quant	61,444	− 0.0127	0.0044	4.52 × 10^–4^
	Wt	Quant	61,443	− 0.0151	0.0053	8.64 × 10^–3^
	WSR	Quant	61,443	0.0133	0.0049	3.34 × 10^–2^
Cardiovascular diseases	Crdmyo	Binary	0 = 60,861 1 = 591	− 0.277	0.0654	1.80 × 10^–2^
Hematological	Plt	Quant	61,423	0.0229	0.0060	3.22 × 10^–4^
Hepatic	AfpAstAltRatio	Quant	61,435	0.0209	0.0060	2.68 × 10^–2^
Nephrotic	Cr	Quant	61,437	− 0.0172	0.0046	6.16 × 10^–5^
	BUN	Quant	61,437	− 0.0213	0.0057	7.18 × 10^–4^
	eGFR	Quant	61,437	0.0020	0.0020	1.15 × 10^–3^
Ophthalmologic	RtDeN	Ordinal	0 = 60,618 1 = 588 2 = 246	0.0900	0.00024	6.11 × 10^–6^

*Std. Err.* standard error, *Fdr* local false discovery rate. Traits with a Fdr < 0.05 were listed

Third, when considering the ethnic differences, the phenotypic associations of rs75776403 in European (from UK Biobank [UKB]) and Japanese (from Biobank Japan [BBJ]) populations were further investigated. Of 2173 traits from Europeans, 22 were found to relate to rs75776403, which including body height (sitting height, Fdr = 6.21 × 10^–10^; standing height, Fdr = 7.46 × 10^–3^), pulmonary functionality (forced expiratory volume in 1-s (FEV1), Fdr = 1.35 × 10^–8^; forced vital capacity (FVC), best measure, Fdr = 1.35 × 10^–8^; FEV1, best measure, Fdr = 3.16 × 10^–8^; FVC, Fdr = 3.16 × 10^–8^; FEV1, predicted percentage, Fdr = 7.09 × 10^–6^; FEV1, predicted, Fdr = 3.22 × 10^–3^), smoke intake (smoking status: never, Fdr = 7.09 × 10^–6^; past tobacco smoking, Fdr = 4.65 × 10^–5^; ever smoked, Fdr = 7.41 × 10^–3^; smoking status: previous, Fdr = 2.40 × 10^–2^), hematological profile (eosinophil count, Fdr = 2.43 × 10^–3^; lymphocyte count, Fdr = 1.45 × 10^–2^), hand grip strength (right, Fdr = 2.93 × 10^–3^), onset age of menarche (Fdr = 2.17 × 10^–2^), beef intake (Fdr = 3.22 × 10^–2^), place of birth (Fdr = 3.22 × 10^–2^), wheezing or whistling in the chest in last year (Fdr = 3.22 × 10^–2^), and the number of depression episodes (Fdr = 3.75 × 10^–2^; Additional file 1: Table S11, top). For the Japanese population, only three out of 229 traits were found to be associated with rs75776403, *i.e.*, height (Fdr = 1.51 × 10^–8^), body weight (Fdr = 5.02 × 10^–3^), and serum creatinine (Fdr = 6.13 × 10^–3^; Additional file 1: Table S11, bottom).

**Fig. 2:**
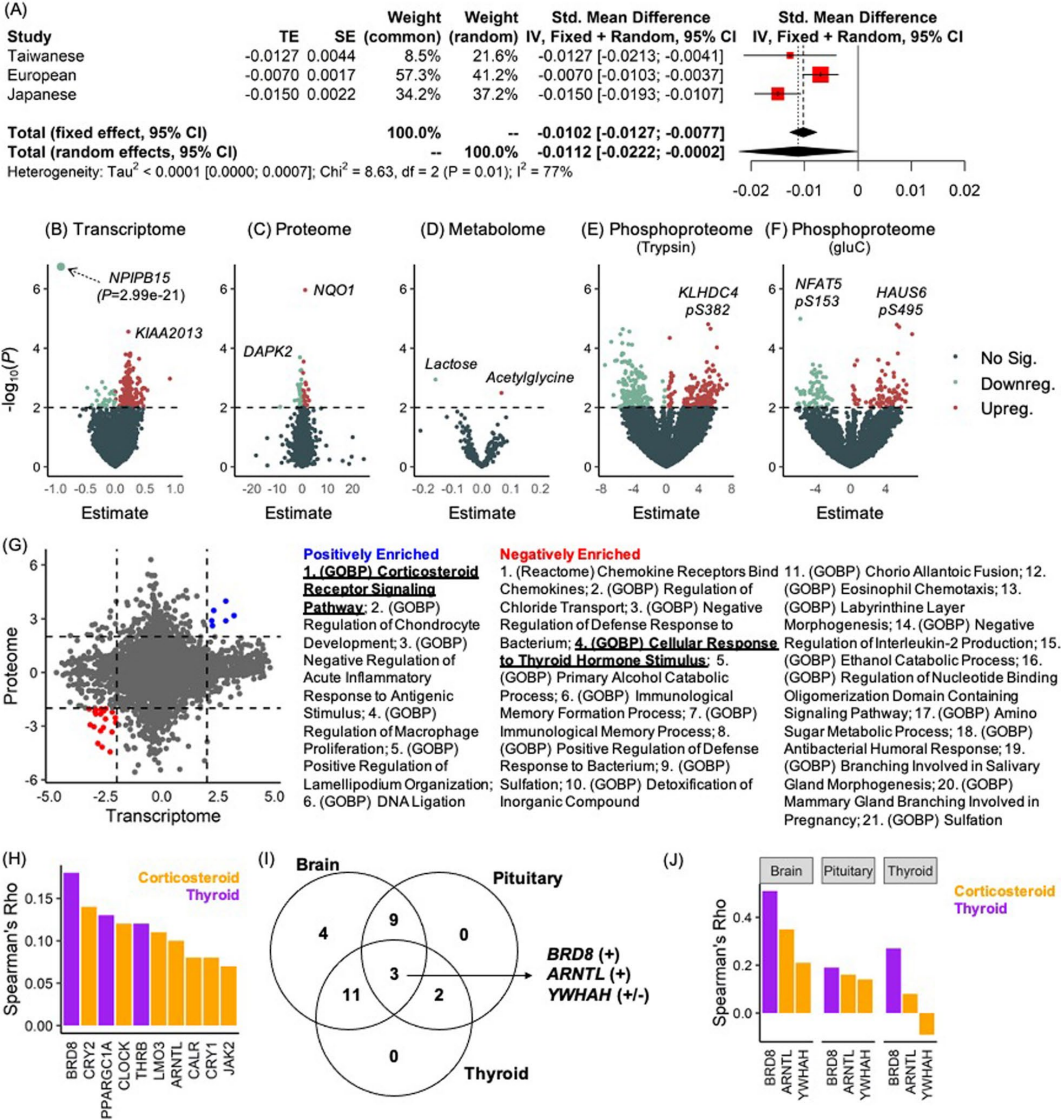
Multiomics profiling of rs75776403. **A** Forest plot showing the meta-analysis statistics of rs75776403 to human body height across ethnicities (Taiwanese, European, and Japanese). Fixed-effect and Knapp-Hartung adjusted random-effect models were both applied. **B**–**F** Volcano plots showing the differential transcriptomic **B** proteomic **C** metabolomic **D**, and phosphoproteomic (trypsin- **E** or gluC-digested **F** analyses to identify molecular features (mRNAs, proteins, metabolites and phosphorylation sites) significantly associated with rs75776403. These features were categorized into up-regulated (red) and down-regulated (pale green) according to estimates (*i.e.*, statistical model coefficient; *x*-axis). Horizontal dashed lines indicate *P* < 0.01 (*y*-axis). **G** Combined analysis of gene-set enrichment analysis (GSEA) results from expression data (*i.e.*, transcriptome and proteome). Dashed lines indicate a scaled weighted statistic (*x*- and *y*-axes) of + 2 or − 2. **H** Bar plot showing genes (*x*-axis) that are associated with *CLEC18A* transcript levels (*P* < 0.05). The strength of the association was quantified by Spearman’s rho statistic (*y*-axis). Genes that are involved in cellular response to thyroid hormone stimulus and corticosteroid receptor signaling are colored in purple and orange, respectively. **I** Venn diagram showing overlapping of *CLEC18A* transcript-associated genes across three tissues (brain, pituitary gland and thyroid). **J** Bar plot showing three genes (*x*-axis) associated with *CLEC18A* transcript levels in all brain, pituitary gland and thyroid tissues (*P* < 0.05). The strength of the association was quantified by Spearman’s rho statistic (*y*-axis). Genes that are implicated in cellular responses to thyroid hormone stimulus and corticosteroid receptor signaling are colored in purple and orange, respectively

Fourth, a trans-ethnic meta-analysis was conducted to estimate the combined effect size of the rs75776403 c.C-to-T variant on the human trait(s) across three populations. Here, only the body height trait was subjected to the meta-analysis because the results passed a phenome-wide significance threshold in all populations (TWB: *β* ± standard error (s.e.) =  − 0.0127 ± 0.0044, Fdr = 4.52 × 10^–4^; UKB: *β* ± s.e. =  − 0.0070 ± 0.0017, Fdr = 7.46 × 10^–3^; BBJ: *β* ± s.e. =  − 0.015 ± 0.0022, Fdr = 1.51 × 10^–8^). Fixed-effect and random-effect models illustrated a combined effect size (*β* ± s.e.) of − 0.0102 ± 0.0013 (*P* = 1.918 × 10^–15^) and − 0.0112 ± 0.0026 (*P* = 0.0485), respectively (Fig. 2A).

### Multiomics profiling of molecular impacts of the CLEC18A c.452C-to-T polymorphism

Since the phenotypic relevance of rs75776403 (*CLEC18A* c.452C-to-T) was elaborated, the next critical question was to know how the genetic variant at this locus impact human traits through perturbing specific molecular or cellular processes. We thus leveraged human cell line-based multiomics (compiling transcriptome, proteome, metabolome, and phosphoproteome) from the Cancer Cell Line Encyclopedia (CCLE) database to conduct the following analyses.

For each molecular feature from the transcriptome and proteome (totals of 19,095 mRNAs and 12,183 proteins), a linear regression model was fitted and associations with rs75776403 were tested by exact permutation. Next, gene set enrichment analysis (GSEA) based on the KEGG, GO BP, and Reactome databases was conducted to identify the potential pathways (and/or gene sets). As a result, we found 278 (251 upregulated and 27 downregulated) and 70 (33 upregulated and 37 downregulated) differentially expressed mRNAs and proteins, respectively (Fig. 2B, C**)**. The GSEA analysis further revealed 392 (365 positively and 27 negatively) and 85 (34 positively and 51 negatively) significantly enriched pathways from mRNAs and proteins, respectively (Additional file 1: Table S12). Intriguingly, the GSEA results pinpointed positive enrichment in the cell cycle, in contrast to negative enrichment in (carbohydrate, amino acid, and lipid) metabolism and immune activation, thus confirming functional consequences (*i.e.*, metabolic or proliferative shifts, and immune deactivation) of *CLEC18A* transcription and/or translation due to the rs75776403 c.C-to-T polymorphism.

Similar analyses were conducted for metabolomic and phosphoproteomic data. For 225 metabolites, we detected two differentially expressed metabolites, including lactose (*β* =  − 0.158, *P* = 1.145 × 10^–3^) and acetylglycine (*β* = 0.067, *P* = 3.223 × 10^–3^; Fig. 2D). For the phosphoproteome (p-site number: 44999 for trypsin and 18,347 for gluC enzymes), 5079 (2526 upregulated and 2553 downregulated) and 2121 (1258 upregulated and 863 downregulated) differentially expressed p-sites were identified for trypsin and gluC, respectively (Fig. 2E, F). The GSEA further revealed eight positively enriched pathways (including epidermal growth factor receptor 1 [EGFR1], glucagon-like peptide-1 [GLP1], phosphoinositide 3-kinase-protein kinase B [PI3K-Akt] signaling, androgen receptor, gastrin, thymic stromal lymphopoietin [TSLP], C–C chemokine receptor type 7 [CCR7], and interleukin 11 [IL-11]; all with *P* ≤ 0.05 and an enrichment score of > 0) to the phosphoproteomic profile associated with the rs75776403 c.C-to-T polymorphism (Additional file 1: Table S13).

### Implications of CLEC18A p.T151M in hormonal regulation

Although these findings imply that metabolic, proliferative and immune-related pathways are governed by CLEC18A p.T151M, it was still unclear whether an upstream regulatory mechanism mediates the molecular and/or cellular processes. To determine these, we combined mRNAs- and proteins-derived GSEA results and identified 27 robust (six positively and 21 negatively enriched) pathways (Fig. 2G and Additional file 1: Fig. S2).

Specifically, we noticed a negatively enriched cellular response to thyroid hormone stimulus (GO ID: 0,097,067) and a positively enriched corticosteroid receptor signaling pathway (GO ID: 0,031,958) associated with rs75776403. Coordinating thyroid hormone with the steroid was found to be essential for growth regulation [23, 24], and abnormal thyroid hormone regulation was reported to influence height in human and animal models [25–27]. Accordingly, we speculated that the significant enrichment of p.T151M-regulated mRNAs and proteins in hormone-related pathways may be related to the rs75776403-associated traits (such as body height) as identified in the PheWAS. To test this, we evaluated the effects of the rs75776403 c.C-to-T polymorphism on mRNA expression levels of seven thyroid hormone axial genes including thyroglobulin (*TG*), thyroid hormone receptor beta (*THRB*), thyroid-stimulating hormone subunit beta (*TSHB*), thyrotropin-releasing hormone (*TRH*), thyroid-stimulating hormone receptor (*TSHR*), thyroid hormone receptor alpha (*THRA*), and thyrotropin-releasing hormone receptor (*TRHR*). Indeed, negative associations with *TG* (*β* =  − 0.14, *P* = 0.0307) and *THRB* (*β* =  − 0.22, *P* = 0.0436) were found (Additional file 1: Table S14). In addition, correlations between the *CLEC18A* mRNA level with these genes were further examined, resulting in significant correlations of *TG* (Spearman’s rho [*ρ*] = 0.12, *P* = 2.20 × 10^–4^), *THRB* (*ρ* = 0.12, *P* = 2.90 × 10^–4^), *TSHR* (*ρ* = 0.13, *P* = 3.43 × 10^–5^), *THRA* (*ρ* = 0.11, *P* = 1.09 × 10^–3^) and *TRHR* (*ρ* = 0.10, *P* = 2.39 × 10^–3^).

Sixteen and 14 genes were respectively identified at the leading edge of cellular responses to the thyroid hormone stimulus (GO ID: 0,097,067) and corticosteroid receptor signaling pathways (GO ID: 0,031,958) (Additional file 1: Table S15). By conducting similar analyses, rs75776403 c.C-to-T polymorphism was found to positively associated with *ARID1A*, *YWHAH*, *CRY1*, *LMO3*, and *JAK2* and that was negatively associated with *THRB* (Additional file 1: Table S16). Moreover, *CLEC18A* mRNA was positively associated with four rs75776403-associated genes (*THRB*, *LMO3*, *CRY1*, and *JAK2*) and other six genes (*BRD8*, *CRY2*, *PPARGC1A*, *CLOCK*, *ARNTL*, and *CALR*; Fig. 2H).

Because hypothalamus-pituitary-thyroid (HPT) axis maintains thyroid hormone homeostasis in humans, correlations of *CLEC18A* mRNA with 30 leading-edge genes in the cellular responses to the thyroid hormone stimulus and corticosteroid receptor signaling pathways were examined within involved (HPT) tissues (brain, pituitary, and thyroid). We found 27 (90.0%), 14 (46.7%), and 16 (53.3%) genes that respectively exhibited an association (*P* < *0.05*) with *CLEC18A* mRNA expression in the brain, pituitary gland, and thyroid (Table 3). Notably, *BRD8*, *ARNTL*, and *YWHAH* were significantly correlated to the *CLEC18A* transcript in three HPT tissues. Importantly, *BRD8* and *ARNTL* showed the consistent effect directions, in contrast to *YWHAH* which showed contrary results among the tissues (Figs. 2I-J). The similar approaches were applied in other thyroid hormone related tissues, such as the adrenal gland, liver, ovary and testis (Additional file 1: Table S17). The data suggested that rs75776403 plays roles in both upstream biosynthesis and downstream hormonal responses regulated by the thyroid hormone.

**Table 3:** Association between *CLEC18A* mRNA level and the (leading edge) genes implicated in the thyroid-stimulated pathway or corticosteroid receptor signaling

	Brain (*N* = 3326)			Pituitary (*N* = 301)			Thyroid (*N* = 812)		
Gene	*ρ*	*r*.^2^	*P*	*ρ*	*r*.^2^	*P*	*ρ*	*r*.^2^	*P*
*BRD8*	0.51	0.26	**2.78 × 10**^**–176**^	0.19	0.04	**9.87 × 10**^**–4**^	0.27	0.07	**3.10 × 10**^**–12**^
*MED1*	0.49	0.24	**1.80 × 10**^**–161**^	0.13	0.02	**0.0286**	− 0.07	0.01	0.0660
*KLF9*	0.39	0.15	**1.65 × 10**^**–96**^	0.06	0.00	0.3288	− 0.05	0.00	0.2190
*CTSL*	0.36	0.13	**2.54 × 10**^**–83**^	0.01	0.00	0.8353	− 0.13	0.02	**5.92 × 10**^**–4**^
*CTSB*	0.32	0.10	**3.00 × 10**^**–63**^	0.04	0.00	0.5400	− 0.12	0.02	**1.46 × 10**^**–3**^
*GAS2L1*	0.31	0.10	**2.55 × 10**^**–61**^	0.24	0.06	**5.31 × 10**^**–5**^	0.05	0.00	0.1653
*KIT*	0.31	0.10	**2.65 × 10**^**–61**^	0.13	0.02	**0.0255**	0.02	0.00	0.5377
*PPARGC1A*	0.31	0.09	**3.73 × 10**^**–59**^	0.04	0.00	0.4553	− 0.04	0.00	0.3089
*GCLM*	0.29	0.09	**6.08 × 10**^**–54**^	0.02	0.00	0.7377	− 0.17	0.03	**9.73 × 10**^**–6**^
*RDX*	0.20	0.04	**9.01 × 10**^**–26**^	0.22	0.05	**1.96 × 10**^**–4**^	− 0.05	0.00	0.2176
*GATA1*	0.17	0.03	**1.71 × 10**^**–19**^	0.08	0.01	0.1979	0.12	0.01	**2.41 × 10**^**–3**^
*CTSS*	0.09	0.01	**1.10 × 10**^**–6**^	0.12	0.01	**0.0471**	− 0.00	0.00	0.9007
*GHSR*	0.08	0.01	**5.17 × 10**^**–5**^	− 0.08	0.01	0.1551	− 0.07	0.00	0.0932
*LMO2*	0.03	0.00	0.1348	0.10	0.01	0.1102	0.01	0.00	0.7788
*CTSH*	− 0.02	0.00	0.3714	0.13	0.02	**0.0320**	0.12	0.01	**2.48 × 10**^**–3**^
*THRB*	0.01	0.00	0.5759	0.15	0.02	**0.0112**	0.10	0.01	**0.0104**
*ARID1A*	0.51	0.26	**7.20 × 10**^**–175**^	0.25	0.06	**3.04 × 10**^**–5**^	− 0.02	0.00	0.6308
*JAK2*	0.46	0.21	**1.73 × 10**^**–136**^	0.09	0.01	0.1476	0.10	0.01	**9.34 × 10**^**–3**^
*CRY1*	0.44	0.20	**3.01 × 10**^**–128**^	0.02	0.00	0.7632	0.09	0.01	**0.0165**
*CLOCK*	0.44	0.20	**1.00 × 10**^**–126**^	0.16	0.03	**6.02 × 10**^**–3**^	0.07	0.00	0.0906
*NR3C1*	0.44	0.19	**2.68 × 10**^**–126**^	0.04	0.00	0.5404	0.09	0.01	**0.0209**
*PPP5C*	0.43	0.18	**3.70 × 10**^**–119**^	0.11	0.01	0.0717	− 0.06	0.00	0.1578
*PER1*	0.41	0.17	**9.34 × 10**^**–110**^	0.13	0.02	**0.0254**	0.07	0.00	0.0804
*CALR*	0.37	0.13	**3.60 × 10**^**–85**^	0.04	0.00	0.5400	− 0.13	0.02	**1.24 × 10**^**–3**^
*CRY2*	0.37	0.13	**3.96 × 10**^**–85**^	0.11	0.01	0.0542	0.11	0.01	**5.13 × 10**^**–3**^
*ARNTL*	0.35	0.13	**5.73 × 10**^**–79**^	0.16	0.03	**5.90 × 10**^**–3**^	0.08	0.01	**0.0352**
*PHB*	0.32	0.11	**5.56 × 10**^**–66**^	0.09	0.01	0.1534	− 0.11	0.01	**4.25 × 10**^**–3**^
*YWHAH*	0.21	0.05	**1.79 × 10**^**–28**^	0.14	0.02	**0.0223**	− 0.09	0.01	**0.0185**
*NEDD4*	0.06	0.00	**1.93 × 10**^**–3**^	0.08	0.01	0.1705	0.08	0.01	**0.0304**
*LMO3*	-0.05	0.00	**4.91 × 10**^**–3**^	0.21	0.04	**4.38 × 10**^**–4**^	0.07	0.00	0.0888

*N* sample size. Genes implicated in thyroid stimulated pathways were labeled in white color; while genes implicated in corticosteroid receptor signaling were labeled in grey color. A *P* value of < 0.05 was highlighted in bold

## Discussion

Despite remarkable advances in genetics, little attention has been paid to whether and how genetic variants impact aspects of molecular and/or cellular features in the pathophysiology of human traits beyond simple susceptibility. Herein, we identified a missense *cis*-eQTL (p.T151M) in CLEC18A that was mainly correlated with anthropometrics (especially body height), nephrotic, and hematological traits (and/or diseases) in Taiwanese, European and Japanese populations. This observation implicates the pleiotropy (associated with more than one trait) of rs75776403 as well as the concept that a genetic polymorphism could exert functional impacts through both regulating gene expressions (as a *cis*-eQTL) and disrupting a critical protein domain (as a missense variant). Focusing on these pathophysiological aspects may provide novel insights regarding genetic epidemiological profiles of the given trait or disease, as well as facilitate precision medicine.

CLEC18A has been linked to host immune defence against dengue viral infection through the C-type lectin-like domain (CTLD) [2]. Serum CLEC18 protein levels were found to be positively correlated with the HBV DNA load, HBsAg levels, and HCV viral loads [3, 4]. Nonetheless, whether CLEC18A is associated with other diseases, or which protein domain is responsible for the physiological correlation, is still unclear. By exploring the functional effects of the rs75776403 c.C-to-T polymorphism, we identified that this variant promotes metabolic and proliferative shifts, as well as immune deactivation in human cells. This finding, therefore, not only elaborates on molecular and/or cellular links to the genetic associations of rs75776403, but also sheds light on the functional roles of CLEC18A in all cell types (such as immune cells) that may be implicated in the associated traits or diseases.

The CAP/SCP/TAPS domain, which is possessed by CLEC18A and other genes, was proposed to bind and transport sterols, acidic glycolipids, and/or acidic phospholipids [28, 29]. Specifically, this domain is directly responsible for packing lipids in the endoplasmic reticulum (ER) into vesicles and secreting them out of cells [30]. Here, rs75776403 p.T151M was shown to disrupt the binding affinity of the CAP/SCP/TAPS domain to the PA and PS (both of which are acidic phospholipids). Notably, further experimental validation is needed to consolidate our findings. Since PA and its derivative, lysophosphatidic acid (lysoPA), may act as extracellular ligands of G protein-coupled receptors (GPCRs) [31], which play pivotal roles in metabolism [32], diminished extracellular exportation of PA due to rs75776403 p.T151M disruption of the CAP/SCP/TAPS domain may lead to less GPCR signaling, and thus perturb homeostasis of metabolites including carbohydrates, amino acids and lipids [32, 33]. It is noteworthy that PA and lysoPA may also act as mitogens. Therefore, CLEC18A p.T151M may lead to the accumulation of free-form PA by either decreasing extracellular exportation of PA or decreasing CLEC18A-bounded PA. These free-form PAs may thus exert mitogenic functions to enhance cell proliferation. Furthermore, PS may also bind to the receptor for advanced glycation end products (RAGE) and affect cell cycle genes implicated in the G_1_/S phase transition [34].

The underlying mechanisms of immune deactivation through CLEC18A p.T151M can be explained as follows. First, PS-recognized receptors share a common feature that promotes the production of anti-inflammatory mediators [34, 35]. Hence, rs75776403 p.T151M may lead to a greater amount of the free form of intracellular PS and contribute to immune inhibition. Second, consistent with speculation that CLEC18A may extract sterols from assembled membranes of pathogens (*e.g.*, dengue virus type 2 [36]) during the intracellular viral replication stage in the ER [1], we observed negative enrichment of immune activation pathways by rs75776403 p.T151M. Third, genes with the CAP/SCP/TAPS domain were proposed to be a Ca^2+^-specific serine protease [37, 38], which is critical for antigen processing and to elicit pathogen-specific immune activation of T cells [39, 40]. rs75776403 p.T151M may thus disrupt immune activation through diminishing the proteolytic activity of CLEC18A. Fourth, PA was implicated in the biogenesis of recycling endosomes (REs) [41]. Since the endosome is critical for innate and adaptive immune function [42, 43], we proposed that CLEC18A p.T151M may cause retention of PA in the ER, thus weakening the integrity of membrane structure and the subcellular function of endosomes. Notably, a decrease in the amount of PA in RE may also affect the recycling/turnover of EGFR [44].

Additionally, we identified a correlation of rs75776403 p.T151M and *CLEC18A* transcripts with the end products of the thyroid hormone biogenesis pathway, thyroglobulin (*TG*) and thyroid hormone receptor (*THRB*). We should notice that the upstream receptors of the pathway, thyrotropin-releasing hormone receptor (TRHR) [45] and thyrotropin receptor (TSHR) [46], are also GPCRs. Thus, loss of PA-binding affinity due to rs75776403 p.T151M may result in loss of synergistic G protein activation. Specifically, thyroid hormones (triiodothyronine (T3), and thyroxine (T4)) are essential for growth, development, and metabolisms [47, 48]. The functions of triiodothyronine is not only in cell proliferation [49–51], branching morphogenesis of the lungs [52], but also in the DNA synthesis, differentiation to osteoblasts, and act on growth plate chondrocytes [53, 54]. Thus, previous findings were in line with our results that the rs75776403 variant significantly associates with the human growth phenotypes.

Moreover, thyroid hormones modulate the overall synthesis of phosphocreatine (PCr) through the regulations for creatine kinase and mitochondrial oxidative phosphorylation [55]. PCr is a protector against cardiac disease, as a meta-analysis revealing that uptake of extra PCr produced lower risks for all cardiac incidences [56]. Thus, it is reasonable to speculate that the rs75776403 p.T151M correlation with lower risk for cardiomyopathy may be due to thyroid hormones-mediated PCr accumulation. Moreover, consistent with our PheWAS results, thyroid hormones were shown to alter several biomarkers related to kidney function (serum levels of creatinine and eGFR) [57] and platelets (mean platelet count, mean platelet volume, and platelet distribution width) [58, 59].

To date, more than 5500 publications and ~ 330 K associations have been elaborated (by the Genome-Wide Association Study Catalog; February 2022), but most results still need to be functionally validated. One of the possible ways to accomplish this is to integrate data from multiple sources. Herein, we leveraged *cis*-eQTL annotations from the GTEx database to confirm the tissue-specific *cis*-regulatory effects of the rs75776403 c.C-to-T polymorphism on mRNA levels of *CLEC18A*. Furthermore, a protein domain simulation and lipid strip test were conducted to confirm that missense rs75776403 p.T151M abruptly affects the lipid-binding ability of the CLEC18A. By further incorporating the rs75776403-correlated multiomics to pinpoint metabolic/proliferative shift and implication in thyroid hormonal regulation, these results elaborate a promising way to link the molecular and/or cellular features of rs75776403 to phenotypic landscape of humans.

## Conclusions

**Fig. 3:**
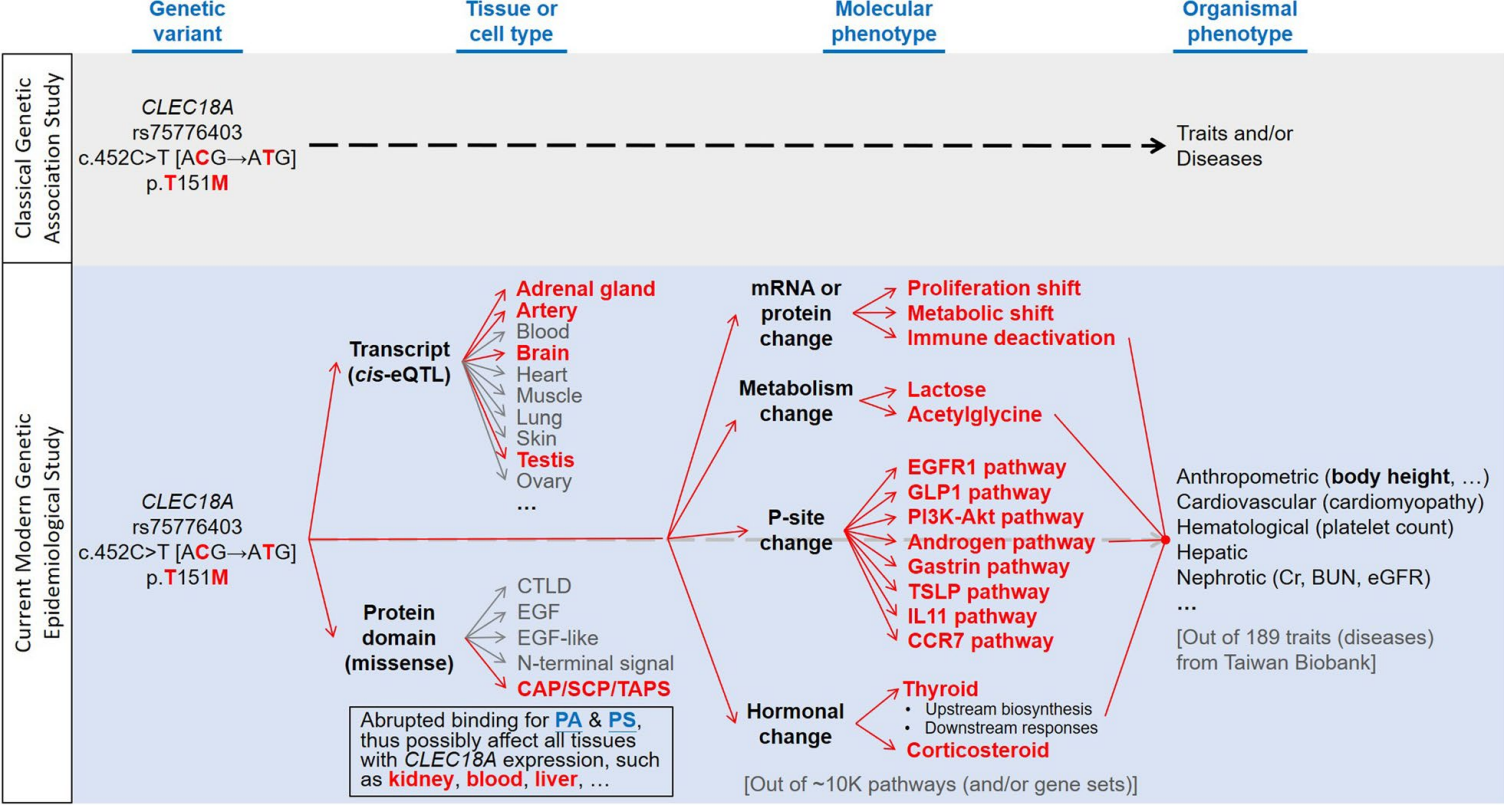
Summary of the current study. A modern genetic epidemiological study parsing molecular and cellular features of *CLEC18A* rs75776403 that contribute to human traits and diseases

The rs75776403 was identified as a crucial missense *cis*-eQTL in *CLEC18A*, and linked the polymorphism to various phenotypes. We demonstrated that CLEC18A binds specifically to two phospholipids, PA and PS via the CAP/SCP/TAPS domain. Defects in the lipid-binding ability were identified in the rs75776403 altered allele (p.M151) carriage. By elaborating *CLEC18A* rs75776403-associated multiomics, CLEC18A has great impact to regulate cellular processes, metabolism, cell cycle, immune activation, thyroid hormone biosynthesis and immune responses. Thus, a single amino acid change T_151_→M_151_ ultimately contributes to the variability of human traits and differential outcome of human diseases. This study demonstrates a promising approach to get insights on the potential impact of genetic polymorphism for precision medicine (Fig. 3).

## Supplementary Information

**Additional file 1: Table S1.** Frequency of non-synonymous variants in CLEC18 genes among different ethnic populations from the data of 1000 Genome Project. **Table S2.** The 48 variants as significant *cis*-expression quantitative trait loci (*cis*-eQTL) for CLEC18 family genes (GTEx Portal). **Table S3**. The *cis*-eQTL annotation of rs75776403. **Figure S1.** Boxplot showing the distribution of *CLEC18A* transcript (mRNA) levels across rs75776403 genotypes. **Table S4.** The 86 quantitative traits (11 phenotype categories) included in this study (*N* = 68,080). **Table S5.** The 24 additional quantitative traits are included in this study. **Table S6.** Association results of rs75776403 to the quantitative traits. **Table S7.** The 84 binary traits (16 phenotype categories) are included in this study. **Table S8.** Association results of rs75776403 and binary traits. **Table S9.** The 19 ordered traits (4 phenotype categories) are included in this study. **Table S10.** Association results of rs75776403 and ordinary traits. **Table S11.** Phenome-wide association study for *CLEC18A* rs75776403 in UK Biobank (UKB) and BioBank Japan (BBJ). **Table S12.** Significantly enriched pathways (and/or gene sets) were identified by gene-set enrichment analysis (GSEA). **Table S13.** Gene-set enrichment analysis (GSEA) results of phosphoproteomics that were associated with rs75776403. **Figure S2.** Pairwise correlation plot showing a gene–gene correlation between genes that are involved in the cellular response to thyroid hormone stimulus (purple) and corticosteroid receptor signaling pathways (orange). *CLEC18A* gene is marked using a red dot. **Table S14.** Association between (A) rs75776403 or (B) *CLEC18A* mRNA level with thyroid hormone axial genes. **Table S15.** Gene set of cellular response to thyroid hormone stimulus and corticosteroid receptor signaling pathway in gene ontology (GO) biological process (BP) database. **Table S16.** Association between rs75776403 and corticosteroid receptor signaling or thyroid-stimulated pathway genes. **Table S17.** Association between *CLEC18A* and thyroid stimulated pathways or corticosteroid receptor signaling genes in adrenal gland, liver, ovary, and testis tissues from GTEx portal.

## Abbreviations

CAP: C-terminal cysteine-rich secretory protein/antigen 5/pathogenesis related-1
*cis*-eQTL: *cis*-Acting expression quantitative trait locus
CLEC18: C-type lectin 18
CRD: Carbohydrate recognition domain
ER: Endoplasmic reticulum
HBV: Hepatitis B virus
SCP: Sperm-coating protein
TAPS: Tpx antigen 5/pathogenesis related-1/Sc7
EGF: Epidermal growth factor
1000G: 1000 Genome
GTEx: Genotype-Tissue Expression
SIFT: Sort Intolerant From Tolerant
PolyPhen-2: Polymorphism Phenotyping v2
CADD: Combined Annotation Dependent Depletion
3D: 3-Dimensional
APBS: Adaptive Poisson-Boltzmann Solver
WT: Wild-type
PIP: Phosphorylated derivative of phosphatidylinositol
BSA: Bovine serum albumin
PBS: Phosphate-buffered saline
h: Hour
IgG: Immunoglobulin G
TWB: Taiwan Biobank
IRB: Institutional Review Board
chr: Chromosome
MAF: Minor allele frequency
HWE: Hardy–Weinberg equilibrium
LD: Linkage disequilibrium
GCTA: Genome-wide Complex Trait Analysis
PC: Principal component
SNP: Single-nucleotide polymorphism
PheWAS: Phenome-wide association study
Fdr: Local false discovery rate
UKB: UK Biobank
BBJ: Biobank Japan
CCLE: Cancer cell line encyclopedia
DepMap: Dependency Map
p-site: Phosphorylation site
GSEA: Gene-set enrichment analysis
KEGG: Kyoto Encyclopedia of Genes and Genomes
GO: Gene ontology
BP: Biological process
PA: Phosphatidic acid
PS: Phosphatidylserine
HPT: Hypothalamus-pituitary-thyroid
CTLD: C-type lectin-like domain
lysoPA: Lysophosphatidic acid
GPCR: G protein-coupled receptor
RE: Recycling endosome
PCr: Phosphocreatinine

## Declarations

### Availability of data and materials

Raw genotype and phenotype data from the Taiwan Biobank are available upon request and application (https://www.biobank.org.tw/about_process.php) for research purposes.
