# Supplementary material for: Human rs75776403 polymorphism links differential phenotypic and clinical outcomes to a CLEC18A p.T151M-driven multiomics
Source: J Biomed Sci. 2022 Jun 18;29:43. doi: 10.1186/s12929-022-00822-1 (PMC9206359; doi:10.1186/s12929-022-00822-1)
Supplement: Supplementary file 1 — Additional file 1: Table S1. Frequency of non-synonymous variants in CLEC18 genes among different ethnic populations from the data of 1000 Genome Project. Table S2. The 48 variants as significant cis-expression quantitative trait loci (cis-eQTL) for CLEC18 family genes (GTEx Portal). Table S3. The cis-eQTL annotation of rs75776403. Figure S1. Boxplot showing the distribution of CLEC18A transcript (mRNA) levels across rs75776403 genotypes. Table S4. The 86 quantitative traits (11 phenotype categories) included in this study (N = 68,080). Table S5. The 24 additional quantitative traits are included in this study. Table S6. Association results of rs75776403 to the quantitative traits. Table S7. The 84 binary traits (16 phenotype categories) are included in this study. Table S8. Association results of rs75776403 and binary traits. Table S9. The 19 ordered traits (4 phenotype categories) are included in this study. Table S10. Association results of rs75776403 and ordinary traits. Table S11. Phenome-wide association study for CLEC18A rs75776403 in UK Biobank (UKB) and BioBank Japan (BBJ). Table S12. Significantly enriched pathways (and/or gene sets) were identified by gene-set enrichment analysis (GSEA). Table S13. Gene-set enrichment analysis (GSEA) results of phosphoproteomics that were associated with rs75776403. Figure S2. Pairwise correlation plot showing a gene–gene correlation between genes that are involved in the cellular response to thyroid hormone stimulus (purple) and corticosteroid receptor signaling pathways (orange). CLEC18A gene is marked using a red dot. Table S14. Association between (A) rs75776403 or (B) CLEC18A mRNA level with thyroid hormone axial genes. Table S15. Gene set of cellular response to thyroid hormone stimulus and corticosteroid receptor signaling pathway in gene ontology (GO) biological process (BP) database. Table S16. Association between rs75776403 and corticosteroid receptor signaling or thyroid-stimulated pathway genes. Table S17. Ass [file 12929_2022_822_MOESM1_ESM.docx]

| **Table S1.** Frequency of non-synonymous variants in CLEC18 genes among different ethnic populations from the data of 1000 Genome Project. | | | | | | | |
| --- | --- | --- | --- | --- | --- | --- | --- |
| **SNP ID** | **Gene** | **Allele** | **Amino acid** | **Frequency** | | | |
|  |  |  |  | **AFR** | **AMR** | **ASN** | **EUR** |
| rs2549089 | *CLEC18A* | C/T | T91I | 0.016 | NA | NA | NA |
| s145695799 | *CLEC18A* | G/A | G99S | 0.022 | NA | NA | NA |
| rs3869427 | *CLEC18A* | T/C | L100P | 0.049 | 0.308 | 0.16 | 0.356 |
| rs2549097 | *CLEC18A* | C/T | A118V | 0.041 | 0.252 | NA | 0.248 |
| rs60310934 | *CLEC18A* | G/A | A137T | 0.016 | NA | NA | NA |
| rs75776403 | *CLEC18A* | C/T | T151M | 0.114 | 0.323 | 0.476 | 0.271 |
| rs148144594 | *CLEC18B* | C/T | R10Q | 0.014 | NA | NA | NA |
| rs147077893 | *CLEC18B* | C/A | G11V | 0.015 | NA | NA | NA |
| rs540258921 | *CLEC18B* | A/G | I91T | NA | 0.075 | 0.032 | 0.058 |
| rs150702800 | *CLEC18C* | C/T | T151M | NA | NA | NA | 0.014 |
| rs199501835 | *CLEC18C* | A/T | Q152L | NA | NA | 0.024 | NA |
| rs538341866 | *CLEC18C* | C/T | R441W | 0.02 | NA | NA | NA |
| AFR: African. AMR: Admixed American. ASN: including East Asian and South Asian. EUR: European. Frequency less than 1% was not shown. | | | | | | | |

| **Table S2.** The 48 variants as significant *cis*-expression quantitative trait loci (*cis*-eQTL) for CLEC18 family genes (GTEx Portal). | | | | | | |
| --- | --- | --- | --- | --- | --- | --- |
| **No.** | **rs ID** | **Gene** | **Ref** | **Alt** | **Position (GRCh38)** | **Function** |
| 1 | rs139813358 | *CLEC18A* | G | C | chr16:69950242 | 5 Prime UTR Variant |
| 2 | rs199537784 | *CLEC18A* | T | C | chr16:69951371 | Missense Variant (L2P) |
| 3 | rs879509093 | *CLEC18A* | C | G | chr16:69952139 | Intron Variant |
| 4 | rs547486672 | *CLEC18A* | C | G | chr16:69952468 | Intron Variant |
| 5 | rs3869409 | *CLEC18A* | C | T | chr16:69952534 | Intron Variant |
| 6 | rs3874521 | *CLEC18A* | C | A | chr16:69952729 | Intron Variant |
| 7 | rs1972034 | *CLEC18A* | C | T | chr16:69952744 | Intron Variant |
| 8 | rs56140825 | *CLEC18A* | A | G | chr16:69952855 | Intron Variant |
| 9 | rs71397999 | *CLEC18A* | G | A | chr16:69953851 | Intron Variant |
| 10 | rs62053340 | *CLEC18A* | C | T | chr16:69953861 | Intron Variant |
| 11 | rs2549097 | *CLEC18A* | T | C | chr16:69954470 | Missense Variant (V118A) |
| 12 | rs75776403 | *CLEC18A* | C | T | chr16:69954569 | Missense Variant (T151M) |
| 13 | rs149909666 | *CLEC18A* | C | G | chr16:69954764 | Intron Variant |
| 14 | rs4985468 | *CLEC18A* | A | C | chr16:69954810 | Intron Variant |
| 15 | rs55862211 | *CLEC18A* | A | C | chr16:69954909 | Intron Variant |
| 16 | rs563347029 | *CLEC18A* | A | G | chr16:69954935 | Intron Variant |
| 17 | rs201787185 | *CLEC18A* | GAT | G | chr16:69955538-69955540 | Intron Variant |
| 18 | rs184167884 | *CLEC18A* | C | T | chr16:69955973 | Intron Variant |
| 19 | rs613775 | *CLEC18A* | G | T | chr16:69956931 | Intron Variant |
| 20 | rs374347529 | *CLEC18A* | G | A | chr16:69963501 | Intron Variant |
| 21 | rs376143349 | *CLEC18A* | T | A | chr16:69963529 | Intron Variant |
| 22 | rs4985471 | *CLEC18A* | A | T | chr16:69964435 | Intron Variant |
| 23 | rs11075748 | *CLEC18A* | G | T | chr16:69964670 | Intron Variant |
| 24 | rs1296870337 | *CLEC18A* | TGGGAGAC | T | chr16:69965313-69965324 | Intron Variant |
| 25 | rs74637212 | *CLEC18A* | C | A | chr16:69965397 | Intron Variant |
| 26 | rs4985383 | *CLEC18A* | C | T | chr16:69966359 | 3 Prime UTR Variant |
| 27 | rs675008 | *CLEC18A* | A | G | chr16:69966888 | 3 Prime UTR Variant |
| 28 | rs3869414 | *CLEC18A* | G | A | chr16:69967518 | 3 Prime UTR Variant |
| 29 | rs376743879 | *CLEC18C* | C | T | chr16:70173545 | 5 Prime UTR Variant |
| 30 | rs77724138 | *CLEC18C* | G | C | chr16:70173617 | 5 Prime UTR Variant |
| 31 | rs561545353 | *CLEC18C* | C | T | chr16:70173840 | Intron Variant |
| 32 | rs1336240724 | *CLEC18C* | G | A | chr16:70174552 | Intron Variant |
| 33 | rs879876181 | *CLEC18C* | G | C | chr16:70174691 | Intron Variant |
| 34 | rs577877333 | *CLEC18C* | T | G | chr16:70176683 | Intron Variant |
| 35 | rs149087931 | *CLEC18C* | G | A | chr16:70177315 | Synonymous Variant (A97A) |
| 36 | rs557404754 | *CLEC18C* | G | C | chr16:70177638 | Intron Variant |
| 37 | rs139634176 | *CLEC18C* | G | A | chr16:70186488 | Missense Variant (E437K) |
| 38 | rs879849227 | *CLEC18B* | G | A | chr16:74408344 | 500B Downstream Variant |
| 39 | rs146555914 | *CLEC18B* | G | C | chr16:74409644 | Non-Coding Transcript Variant |
| 40 | rs201089329 | *CLEC18B* | A | G | chr16:74415496 | Intron Variant |
| 41 | rs576697401 | *CLEC18B* | G | A | chr16:74416949 | Intron Variant |
| 42 | rs148026772 | *CLEC18B* | C | T | chr16:74419208 | Intron Variant |
| 43 | rs748151773 | *CLEC18B* | T | C | chr16:74419922 | Intron Variant |
| 44 | rs145599906 | *CLEC18B* | G | A | chr16:74420093 | Intron Variant |
| 45 | rs556105693 | *CLEC18B* | G | C | chr16:74420159 | Intron Variant |
| 46 | rs200551840 | *CLEC18B* | C | A | chr16:74421292 | Non-Coding Transcript Variant |
| 47 | rs72791067 | *CLEC18B* | G | C | chr16:74421393 | Non-Coding Transcript Variant |
| 48 | rs4888232 | *CLEC18B* | G | A | chr16:74421563 | Intron Variant |

| **Table S3.** The *cis*-eQTL annotation of rs75776403. | | | | | |
| --- | --- | --- | --- | --- | --- |
| **Variant** | **Gencode ID** | **Gene** | **P Value** | **NES** | **Tissue** |
| rs75776403 | ENSG00000157322.17 | *CLEC18A* | 2.70×10^-17^ | -0.66 | Testis |
|  |  |  | 8.20×10^-15^ | 0.39 | Artery - Tibial |
|  |  |  | 3.10×10^-10^ | 0.46 | Artery - Aorta |
|  |  |  | 3.70×10^-9^ | 0.49 | Artery - Coronary |
|  |  |  | 1.40×10^-5^ | 0.51 | Brain - Cortex |
|  |  |  | 3.20×10^-5^ | 0.45 | Brain - Caudate (basal ganglia) |
|  |  |  | 9.50×10^-5^ | -0.39 | Adrenal Gland |
| NES, normalized effect size. | | | | | |


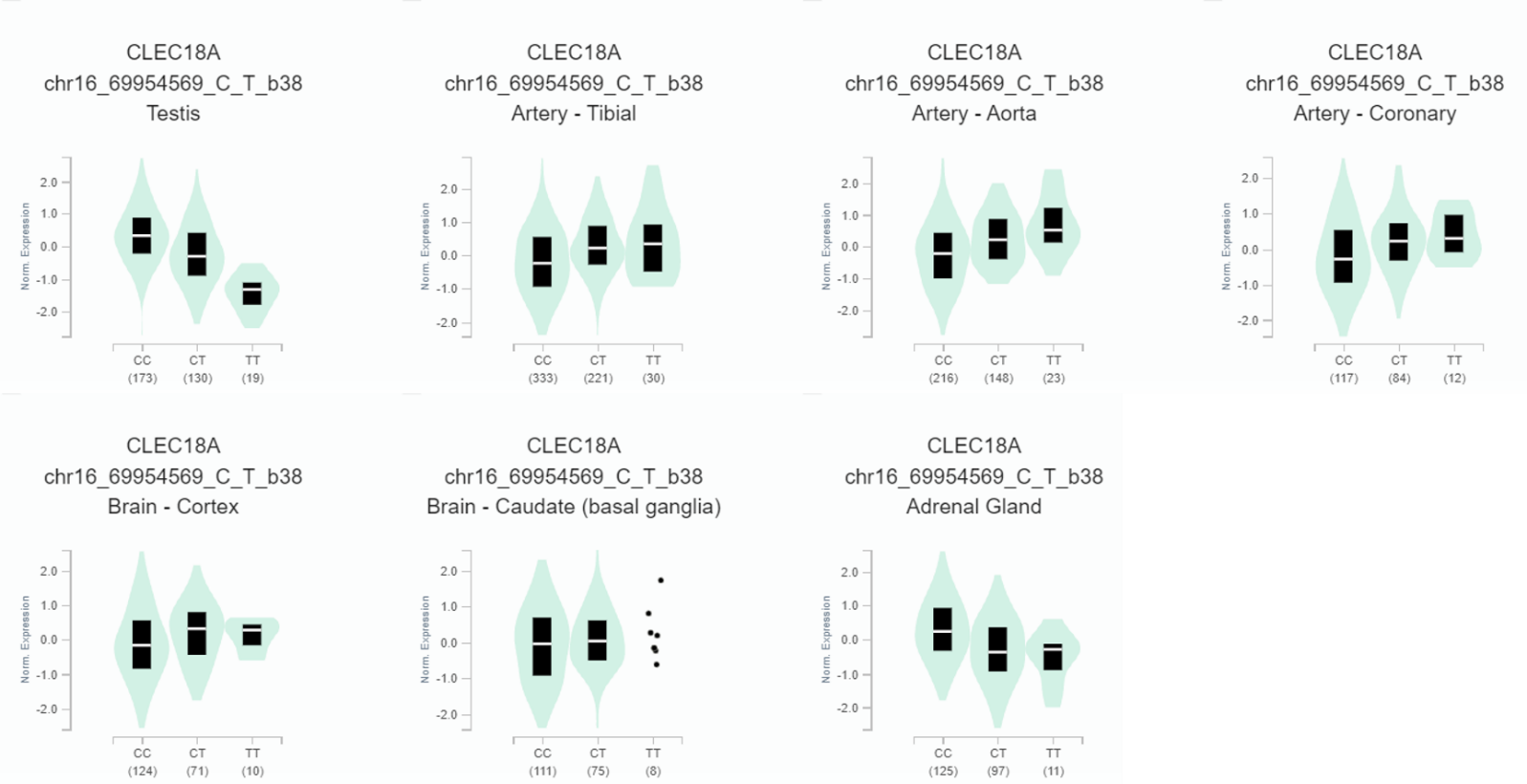


**Fig. S1.** Boxplot showing the distribution of *CLEC18A* transcript (mRNA) levels across rs75776403 genotypes.

| **Table S4.** The 86 quantitative traits (11 phenotype categories) included in this study (*N*=68,080). | | | | | | |
| --- | --- | --- | --- | --- | --- | --- |
| **No.** | **Category** | **Trait (Unit)** | **Abbr.** | **Avg.** | **s.d.** | **Note** |
| 1 | Anthropometric | Height (cm) | Ht | 161.16 | 8.08 |  |
| 2 |  | Weight (kg) | Wt | 63.03 | 12.46 |  |
| 3 |  | Body fat (%) | BF | 29.19 | 7.39 |  |
| 4 |  | Waist circumference (cm) | WC | 83.08 | 10.20 |  |
| 5 |  | Hip circumference (cm) | HC | 95.88 | 7.10 |  |
| 6 |  | Body mass index | BMI | 24.17 | 3.78 | Wt/(Ht/100)^2^ |
| 7 |  | Corpulence index | CI | 15.02 | 2.40 | Wt/(Ht/100)^3^ |
| 8 |  | Waist-to-stature ratio | WSR | 1.34 | 0.15 | WC/Ht |
| 9 |  | Waist-to-hip ratio | WHR | 0.87 | 0.07 | WC/HC |
| 10 |  | Body adiposity index | BAI | 29.01 | 4.15 | HC/[(Ht/100)×($\sqrt{Ht/100}$)]-18 |
| 11 |  | BMI-adjusted WHR | BMIAdjWHR | 0.00 | 0.06 |  |
| 12 |  | BMI-adjusted WC | BMIAdjWC | -0.10 | 5.43 |  |
| 13 |  | BMI-adjusted HC | BMIAdjHC | -0.05 | 3.88 |  |
| 14 |  | Height-adjusted BMI | HtAdjBMI | -0.01 | 3.76 |  |
| 15 | Cardiac | Systolic blood pressure (mmHg) | Sys | 119.69 | 18.06 |  |
| 16 |  | Diastolic blood pressure (mmHg) | Dias | 73.26 | 10.90 |  |
| 17 |  | Heartbeat speed (per 30 sec) | Heartbeat | 35.34 | 4.59 |  |
| 18 |  | Pulse pressure (mmHg) | Pulse | 46.42 | 11.82 | Sys-Dias |
| 19 |  | Systolic-to-diastolic blood pressure ratio | SysDiasRatio | 1.64 | 0.17 | Sys/Dias |
| 20 |  | Diastolic-to-pulse pressure ratio | DiasPulseRatio | 1.66 | 0.44 | Dias/Pulse |
| 21 | Gynecological | Age of menopause (y/o) | Menps | 49.52 | 4.94 | Female-only (for male, coded as missing) |
| 22 |  | Age of menarche (y/o) | Menrc | 13.32 | 1.48 | Female-only (for male, coded as missing) |
| 23 | Habitual | Alcohol intake (y/o) | DRK_y | 17.88 | 12.69 | Alcohol intake years |
| 24 |  | Nut intake | Nut_DsYr | 115.85 | 179.69 | (Intake year)×(Daily dose)^2^ [Daily dose code: 1=less than 10/day; 2=10~20/day; 3=21~30/day; 4=31~40/day; 5=more than 40/day] |
| 25 |  | Smoke intake | SMK_PkYr | 19.61 | 25.14 | (Cigarette package/day)×(Smoking year) |
| 26 | Hematological | Red blood cell count (million/uL) | RBC | 4.72 | 0.51 |  |
| 27 |  | White blood cell count (1000/uL) | WBC | 5.82 | 1.61 |  |
| 28 |  | Platelet count (1000/uL) | Plt | 243.26 | 61.15 |  |
| 29 |  | Hemoglobin (g/dL) | Hb | 13.63 | 1.57 |  |
| 30 |  | Hematocrit (%) | Hct | 41.19 | 4.09 |  |
| 31 |  | Hct-to-Hb ratio | HctHbRatio | 3.03 | 0.15 | Hct/Hb |
| 32 | Hepatic | Total bilirubin (mg/dL) | TBil | 0.66 | 0.28 |  |
| 33 |  | Serum albumin (g/dL) | Alb | 4.50 | 0.23 |  |
| 34 |  | Aspartate aminotransferase (U/L) | AST | 25.01 | 12.47 |  |
| 35 |  | Alanine aminotransferase (U/L) | ALT | 23.27 | 19.56 |  |
| 36 |  | Gamma-glutamyl transferase (U/L) | GGT | 23.80 | 30.92 |  |
| 37 |  | Alpha fetoprotein (ng/mL) | AFP | 3.42 | 11.57 |  |
| 38 |  | AST-to-ALT ratio | AstAltRatio | 1.29 | 0.47 | AST/ALT |
| 39 |  | GGT-to-ALT ratio | GgtAltRatio | 1.10 | 0.86 | GGT/ALT |
| 40 |  | AFP-to-transaminase ratio | AfpAstAltRatio | 0.01 | 0.04 | AFP/(AST×ALT) |
| 41 | Metabolic | Glycated hemoglobin (%) | HbA1c | 5.79 | 0.80 |  |
| 42 |  | Fasting glucose (mg/dL) | FGlu | 95.74 | 20.54 |  |
| 43 |  | BMI-adjusted FGlu | BMIAdjFGlu | -0.12 | 20.12 |  |
| 44 |  | FGlu-to-HbA1c ratio | FGluHbA1cRatio | 16.49 | 1.75 | FGlu/HbA1c |
| 45 |  | Total cholesterol (mg/dL) | TCho | 196.16 | 35.73 |  |
| 46 |  | Triglyceride (mg/dL) | TG | 114.60 | 94.10 |  |
| 47 |  | High density lipoprotein cholesterol (mg/dL) | HDLc | 54.88 | 13.46 |  |
| 48 |  | Low density lipoprotein cholesterol (mg/dL) | LDLc | 120.81 | 31.63 |  |
| 49 |  | TG-to-HDLc ratio | TgHDLcRatio | 2.43 | 2.94 | TG/HDLc |
| 50 |  | TCho-to-HDLc ratio | TChoHDLcRatio | 3.75 | 1.04 | TCho/HDLc |
| 51 | Nephrotic | Blood urea nitrogen (mg/dL) | BUN | 13.07 | 3.91 |  |
| 52 |  | Creatinine (mg/dL) | Cr | 0.71 | 0.30 |  |
| 53 |  | Uric acid (mg/dL) | UA | 5.37 | 1.40 |  |
| 54 |  | Microalbumin (mg/L) | mAlb | 29.45 | 182.36 |  |
| 55 |  | Estimated glomerular filtration rate | eGFR | 5.07 | 0.36 | 186×Cr^-1.154^×Age^-0.203^× [0.742×S]; where S=0 for male and S=1 for female |
| 56 |  | UA-to-Cr ratio | UaCrRatio | 7.89 | 1.96 | UA/Cr |
| 57 |  | BUN-to-Cr ratio | BunCrRatio | 19.36 | 5.93 | BUN/Cr |
| 58 |  | mAlb-to-Cr ratio | mAlbCrRatio | 38.63 | 206.36 | mAlb/Cr |
| 59 | Orthopedic | Stiffness index | SI | 90.90 | 17.28 |  |
| 60 |  | T score | T | -0.42 | 1.62 |  |
| 61 |  | Z score | Z | 0.87 | 1.48 |  |
| 62 |  | Speed of sound (m/sec) | SOS | 1556.20 | 34.14 |  |
| 63 |  | Broadband ultrasound attenuation (dB/MHz) | BUA | 114.14 | 15.48 |  |
| 64 | Pulmonary | Vital capacity (L) | VC | 2.83 | 0.78 |  |
| 65 |  | Tidal volume (L) | TV | 0.77 | 0.41 |  |
| 66 |  | Expiratory reserve volume (L) | ERV | 0.86 | 0.46 |  |
| 67 |  | Inspiratory reserve volume (L) | IRV | 1.21 | 0.55 |  |
| 68 |  | Inspiratory capacity (L) | IC | 1.98 | 0.64 | IRV+TRV |
| 69 |  | VC-to-Ht ratio | VcHtRatio | 1.74 | 0.42 | VC/Ht |
| 70 |  | Forced VC (L) | FVC | 2.73 | 0.78 |  |
| 71 |  | Forced EV in 1 sec (L) | FEV1 | 2.22 | 0.79 |  |
| 72 |  | FEV1-to-FVC ratio | Fev1FvcRatio | 81.12 | 17.14 | (FEV1/FVC)×100 |
| 73 |  | FEV1-to-VC ratio | Fev1VcRatio | 78.76 | 18.88 | (FEV1/VC)×100 |
| 74 |  | Mean maximal flow (L/sec) | MMF | 2.68 | 1.31 |  |
| 75 |  | Peak expiratory flow (L/sec) | PEF | 4.31 | 2.25 |  |
| 76 |  | 25% Forced expiratory flow (L/sec) | FEF25 | 4.08 | 2.16 |  |
| 77 |  | 50% FEF (L/sec) | FEF50 | 3.06 | 1.54 |  |
| 78 |  | 75% FEF (L/sec) | FEF75 | 1.51 | 0.80 |  |
| 79 |  | FEF75-to-Ht ratio (L/sec/m) | Fef75HtRatio | 0.93 | 0.47 | FEF75/Ht |
| 80 |  | Extrapolated volume-to-FVC ratio (%) | EvFvcRatio | 4.74 | 2.34 | (Extrapolate volume/FVC)×100 |
| 81 |  | Forced inspiratory volume in 1 sec-to-FVC ratio (%) | Fiv1FvcRatio | 86.84 | 18.29 | Forced inspiratory volume in 1 sec/FVC |
| 82 | Virological | HCV antibody | AtHCVAb | 1.81 | 22.81 |  |
| 83 |  | Hepatitis B surface antigen | HBsAg | 377.23 | 1480.53 |  |
| 84 |  | Hepatitis B e antigen | HBeAg | 5.53 | 97.44 |  |
| 85 |  | Hepatitis B surface antibody (IU/L) | AtHBsAb | 273.19 | 368.55 |  |
| 86 |  | Hepatitis B core antibody | AtHBcAb | 0.67 | 0.94 |  |
| The 86 original traits queried from the TWB project are highlighted in black color and the 24 derived clinical traits are highlighted in blue. Abbr., abbreviation of each trait used in this study; Avg., average; s.d., standard deviation. The average and standard deviation of each trait were calculated using pre-normalized data. | | | | | | |

| **Table S5.** The 24 additional quantitative traits are included in this study. | | | | | | | |  |
| --- | --- | --- | --- | --- | --- | --- | --- | --- |
| **No.** | **Category** | **Trait (Unit)** | **Abbr.** | **Associated clinical trait(s)** | **Pubmed ID** | **Population** |  |  |
| 1 | Anthropometric | Body mass index | BMI | Diabetes, hypertension, age of puberty, nervousness/anxiety traits | 30707692 | European |  |  |
| 2 |  | Corpulence (Ponderal) index | CI | A better proxy for adiposity than BMI in infants and adults | 28505241 | European (Non-Hispanic) |  |  |
| 3 |  | Waist-to-stature ratio | WSR | Cardiovascular risk factors and related health conditions | 14599732 | East Asian |  |  |
|  |  |  |  | Early health risk | 26975935 | Mainly European (>85%) |  |  |
| 4 |  | Waist-to-hip ratio | WHR | All‐cause mortality and severe obstructive sleep apnoea syndrome | 19639001 | - |  |  |
| 5 |  | Body adiposity index | BAI | Obesity and cardiovascular disease (CVD) | 25583657 | Colombian |  |  |
| 6 |  | BMI-adjusted WHR | BMIAdjWHR | Type 2 diabetes and coronary heart disease | 28196256 | European |  |  |
| 7 |  | BMI-adjusted WC | BMIAdjWC | All-cause, CVD, and cancer mortality | 18362231 | Mainly Caucasian (97%) |  |  |
|  |  |  |  | All‐cause mortality | 20696950 | Mainly Caucasian (97%) |  |  |
| 8 |  | BMI-adjusted HC | BMIAdjHC | Metabolic abnormalities | 21091063 | East Asian |  |  |
|  |  |  |  | Type 2 diabetes | 12716671 | Caucasian |  |  |
| 9 |  | Height-adjusted BMI | HtAdjBMI | Individual growth trajectory | 30368858 | Caucasian |  |  |
| 10 | Cardiac | Pulse pressure (mmHg) | Pulse | Cardiovascular death | 2522417 | Caucasian |  |  |
|  |  |  |  | Coronary heart disease, myocardial infarction, and heart failure (normotensive and hypertensive) | 10421594, 7629401, 10029125 | Mainly Caucasian |  |  |
| 11 |  | Systolic-to-diastolic blood pressure ratio | SysDiasRatio | Renal resistive index | 31586124 | East Asian |  |  |
| 12 |  | Diastolic-to-pulse pressure ratio | DiasPulseRatio | Diurnal rhythm of blood pressure | 25150476, 8347311 | - |  |  |
| 13 | Hematological | Hct-to-Hb ratio | HctHbRatio | Alpha-thalassemias | 24050107 | East Asian |  |  |
|  |  |  |  | Polycythemia vera | 34738212 | Caucasian |  |  |
| 14 | Hepatic | AST-to-ALT ratio | AstAltRatio | Nonalcoholic steatohepatitis and alcoholic liver disease | 10201476 | - |  |  |
|  |  |  |  | Advanced alcoholic liver disease | 15208167 | Caucasian |  |  |
|  |  |  |  | Arterial stiffness | 30509277 | East Asian |  |  |
|  |  |  |  | Type 2 diabetes mellitus | 34819745 | East Asian |  |  |
| 15 |  | GGT-to-ALT ratio | GgtAltRatio | Vascular invasion | 34583704 | East Asian |  |  |
| 16 |  | AFP-to-transaminase ratio | AfpAstAltRatio | Hepatocellular carcinoma | 31027143 | East Asian |  |  |
| 17 | Metabolic | BMI-adjusted FGlu | BMIAdjFGlu | - | 28548082 | Caucasian |  |  |
| 18 |  | FGlu-to-HbA1c ratio | FGluHbA1cRatio | All-cause mortality | 31263559 | East Asian |  |  |
|  |  |  |  | G6PD deficiency | 32156605 | East Asian |  |  |
| 19 |  | TG-to-HDLc ratio | TgHDLcRatio | Insulin resistance | 20019684 | Caucasian |  |  |
|  |  |  |  | Extensive coronary disease | 18719750 | - |  |  |
|  |  |  |  | Ischemic Heart Disease | 34490378 | East Asian |  |  |
|  |  |  |  | Cardiovascular events | 28864368 | East Asian |  |  |
| 20 |  | TCho-to-HDLc ratio | TChoHDLcRatio | Ischemic Heart Disease | 11732933 | Caucasian |  |  |
| 21 | Nephrotic | Estimated glomerular filtration rate | eGFR | - | - | - |  |  |
| 22 |  | UA-to-Cr ratio | UaCrRatio | Outcome in chronic obstructive pulmonary disease | 17294336 | - |  |  |
| 23 |  | BUN-to-Cr ratio | BunCrRatio | Inherited disorders of purine metabolism | 5678000 | - |  |  |
|  |  |  |  | Metabolic syndrome | 32332681 | East Asian |  |  |
| 24 |  | mAlb-to-Cr ratio | mAlbCrRatio | Microalbuminuria | 11912263 | Non-Hispanic whites, non-Hispanic blacks and Mexican Americans |  |  |
| Abbr., abbreviation of each trait used in this study. | | | | | | | | |

| **Table S6.** Association results of rs75776403 to the quantitative traits. | | | | | | | | |
| --- | --- | --- | --- | --- | --- | --- | --- | --- |
| **Traits** | **Beta** | **SE** | **SumSq** | **AvgSq** | **FStat** | **PVal** | **PExtreme** | **PBonf** |
| Nephr:Cr | -1.51E-02 | 4.54E-03 | 1.17E+01 | 1.17E+01 | 2.26E+01 | 1.96E-06 | 1.96E-06 | 1.73E-04 |
| Anthr:Ht | -1.24E-02 | 4.29E-03 | 1.02E+01 | 1.02E+01 | 2.21E+01 | 2.66E-06 | 2.66E-06 | 2.34E-04 |
| Blood:Plt | 2.00E-02 | 5.94E-03 | 1.70E+01 | 1.70E+01 | 1.92E+01 | 1.21E-05 | 1.21E-05 | 1.06E-03 |
| Nephr:BUN | -1.84E-02 | 5.70E-03 | 1.44E+01 | 1.44E+01 | 1.76E+01 | 2.76E-05 | 2.76E-05 | 2.43E-03 |
| Anthr:Wt | -1.51E-02 | 5.19E-03 | 1.13E+01 | 1.13E+01 | 1.66E+01 | 4.59E-05 | 4.59E-05 | 4.04E-03 |
| Nephr:eGFR | 1.01E-03 | 2.02E-03 | 1.69E+00 | 1.69E+00 | 1.64E+01 | 5.02E-05 | 5.02E-05 | 4.42E-03 |
| Hepat:AfpAstAltRatio | 1.95E-02 | 5.97E-03 | 1.08E+01 | 1.08E+01 | 1.20E+01 | 5.27E-04 | 5.27E-04 | 4.64E-02 |
| Pulm:VC | -1.53E-02 | 5.00E-03 | 4.92E+00 | 4.92E+00 | 1.13E+01 | 7.93E-04 | 7.93E-04 | 6.98E-02 |
| Pulm:FVC | -1.46E-02 | 4.92E-03 | 4.34E+00 | 4.34E+00 | 1.02E+01 | 1.38E-03 | 1.38E-03 | 1.21E-01 |
| Anthr:WC | -1.29E-02 | 5.83E-03 | 8.73E+00 | 8.73E+00 | 1.02E+01 | 1.40E-03 | 1.40E-03 | 1.23E-01 |
| Hepat:AFP | 1.99E-02 | 5.61E-03 | 8.06E+00 | 8.06E+00 | 1.02E+01 | 1.41E-03 | 1.41E-03 | 1.24E-01 |
| Blood:Hb | -8.83E-03 | 4.81E-03 | 5.77E+00 | 5.77E+00 | 9.91E+00 | 1.65E-03 | 1.65E-03 | 1.45E-01 |
| Anthr:WSR | 1.08E-02 | 4.83E-03 | 5.59E+00 | 5.59E+00 | 9.51E+00 | 2.04E-03 | 2.04E-03 | 1.80E-01 |
| Pulm:VcHtRatio | -1.54E-02 | 5.31E-03 | 4.65E+00 | 4.65E+00 | 9.44E+00 | 2.12E-03 | 2.12E-03 | 1.87E-01 |
| Anthr:HC | -1.48E-02 | 6.12E-03 | 8.15E+00 | 8.15E+00 | 8.66E+00 | 3.25E-03 | 3.25E-03 | 2.86E-01 |
| Gyn:Menrc | 2.15E-02 | 7.12E-03 | 6.89E+00 | 6.89E+00 | 8.20E+00 | 4.20E-03 | 4.20E-03 | 3.70E-01 |
| Bone:Z | -1.71E-02 | 6.31E-03 | 6.92E+00 | 6.92E+00 | 7.03E+00 | 8.03E-03 | 8.03E-03 | 7.07E-01 |
| Blood:Hct | -7.34E-03 | 4.98E-03 | 4.06E+00 | 4.06E+00 | 6.51E+00 | 1.07E-02 | 1.07E-02 | 9.42E-01 |
| Bone:SI | -1.54E-02 | 5.74E-03 | 4.86E+00 | 4.86E+00 | 5.96E+00 | 1.47E-02 | 1.47E-02 | 1.00E+00 |
| Hepat:AST | -1.14E-02 | 5.86E-03 | 4.87E+00 | 4.87E+00 | 5.63E+00 | 1.77E-02 | 1.77E-02 | 1.00E+00 |
| Anthr:BMI | -1.09E-02 | 6.07E-03 | 4.54E+00 | 4.54E+00 | 4.90E+00 | 2.68E-02 | 2.68E-02 | 1.00E+00 |
| Anthr:WHR | -5.92E-03 | 5.61E-03 | 3.60E+00 | 3.60E+00 | 4.55E+00 | 3.29E-02 | 3.29E-02 | 1.00E+00 |
| Anthr:BMIAdjWC | -5.56E-03 | 5.99E-03 | 4.09E+00 | 4.09E+00 | 4.54E+00 | 3.32E-02 | 3.32E-02 | 1.00E+00 |
| Nephr:UA | -6.36E-03 | 5.37E-03 | 3.03E+00 | 3.03E+00 | 4.18E+00 | 4.08E-02 | 4.08E-02 | 1.00E+00 |
| Nephr:mAlbCrRatio | 1.08E-02 | 6.13E-03 | 3.75E+00 | 3.75E+00 | 3.97E+00 | 4.62E-02 | 4.62E-02 | 1.00E+00 |
| Blood:RBC | -9.40E-03 | 5.42E-03 | 2.91E+00 | 2.91E+00 | 3.94E+00 | 4.72E-02 | 4.72E-02 | 1.00E+00 |
| Blood:WBC | 1.14E-02 | 6.19E-03 | 3.61E+00 | 3.61E+00 | 3.75E+00 | 5.28E-02 | 5.28E-02 | 1.00E+00 |
| Hepat:ALT | -8.89E-03 | 5.82E-03 | 3.14E+00 | 3.14E+00 | 3.69E+00 | 5.49E-02 | 5.49E-02 | 1.00E+00 |
| Pulm:FEV1 | -1.07E-02 | 5.71E-03 | 1.98E+00 | 1.98E+00 | 3.46E+00 | 6.28E-02 | 6.28E-02 | 1.00E+00 |
| Bone:T | -1.53E-02 | 5.83E-03 | 2.90E+00 | 2.90E+00 | 3.46E+00 | 6.29E-02 | 6.29E-02 | 1.00E+00 |
| Anthr:BMIAdjHC | -9.09E-03 | 6.03E-03 | 3.08E+00 | 3.08E+00 | 3.37E+00 | 6.64E-02 | 6.64E-02 | 1.00E+00 |
| Anthr:HtAdjBMI | -9.38E-03 | 6.15E-03 | 3.00E+00 | 3.00E+00 | 3.15E+00 | 7.58E-02 | 7.58E-02 | 1.00E+00 |
| Nephr:UaCrRatio | 8.32E-03 | 6.20E-03 | 2.76E+00 | 2.76E+00 | 2.86E+00 | 9.09E-02 | 9.09E-02 | 1.00E+00 |
| Bone:BUA | -2.76E-02 | 1.17E-02 | 2.32E+00 | 2.32E+00 | 2.81E+00 | 9.36E-02 | 9.36E-02 | 1.00E+00 |
| Blood:HctHbRatio | 4.77E-03 | 5.86E-03 | 2.12E+00 | 2.12E+00 | 2.45E+00 | 1.17E-01 | 1.17E-01 | 1.00E+00 |
| Hepat:GGT | -6.59E-03 | 5.80E-03 | 1.95E+00 | 1.95E+00 | 2.30E+00 | 1.29E-01 | 1.29E-01 | 1.00E+00 |
| Pulm:IC | -7.28E-03 | 6.08E-03 | 1.35E+00 | 1.35E+00 | 2.28E+00 | 1.31E-01 | 1.31E-01 | 1.00E+00 |
| Metab:HbA1c | -5.45E-03 | 5.79E-03 | 1.81E+00 | 1.81E+00 | 2.15E+00 | 1.43E-01 | 1.43E-01 | 1.00E+00 |
| Pulm:PEF | -8.13E-03 | 6.69E-03 | 1.27E+00 | 1.27E+00 | 1.62E+00 | 2.03E-01 | 2.03E-01 | 1.00E+00 |
| Pulm:ERV | -9.10E-03 | 7.15E-03 | 1.08E+00 | 1.08E+00 | 1.32E+00 | 2.51E-01 | 2.51E-01 | 1.00E+00 |
| Hepat:AstAltRatio | 5.34E-03 | 5.85E-03 | 1.11E+00 | 1.11E+00 | 1.28E+00 | 2.57E-01 | 2.57E-01 | 1.00E+00 |
| Viro:AtHBcAb | 5.44E-03 | 4.70E-03 | 6.38E-01 | 6.38E-01 | 1.15E+00 | 2.84E-01 | 2.84E-01 | 1.00E+00 |
| Anthr:CI | -6.69E-03 | 6.20E-03 | 1.09E+00 | 1.09E+00 | 1.12E+00 | 2.89E-01 | 2.89E-01 | 1.00E+00 |
| Nephr:mAlb | 6.83E-03 | 6.22E-03 | 1.01E+00 | 1.01E+00 | 1.04E+00 | 3.08E-01 | 3.08E-01 | 1.00E+00 |
| Anthr:BMIAdjWHR | -7.46E-06 | 5.74E-03 | 8.28E-01 | 8.28E-01 | 1.00E+00 | 3.17E-01 | 3.17E-01 | 1.00E+00 |
| Bone:SOS | -1.19E-03 | 1.18E-02 | 8.26E-01 | 8.26E-01 | 9.69E-01 | 3.25E-01 | 3.25E-01 | 1.00E+00 |
| Pulm:IRV | -4.44E-03 | 6.60E-03 | 6.24E-01 | 6.24E-01 | 8.92E-01 | 3.45E-01 | 3.45E-01 | 1.00E+00 |
| Pulm:FEF25 | -6.02E-03 | 6.76E-03 | 6.60E-01 | 6.60E-01 | 8.24E-01 | 3.64E-01 | 3.64E-01 | 1.00E+00 |
| Pulm:Fev1VcRatio | 4.52E-03 | 7.52E-03 | 7.78E-01 | 7.78E-01 | 7.86E-01 | 3.75E-01 | 3.75E-01 | 1.00E+00 |
| Gyn:Menps | 1.14E-02 | 1.00E-02 | 6.80E-01 | 6.80E-01 | 7.80E-01 | 3.77E-01 | 3.77E-01 | 1.00E+00 |
| Viro:eAgSeroClRate | 9.40E-02 | 2.38E-01 | 8.19E-01 | 8.19E-01 | 7.95E-01 | 3.77E-01 | 3.77E-01 | 1.00E+00 |
| Nephr:BunCrRatio | -4.82E-03 | 5.43E-03 | 3.97E-01 | 3.97E-01 | 5.35E-01 | 4.64E-01 | 4.64E-01 | 1.00E+00 |
| Pulm:TV | -4.89E-03 | 7.49E-03 | 4.45E-01 | 4.45E-01 | 4.93E-01 | 4.83E-01 | 4.83E-01 | 1.00E+00 |
| BP:Heartbeat | -6.09E-03 | 6.19E-03 | 4.34E-01 | 4.34E-01 | 4.51E-01 | 5.02E-01 | 5.02E-01 | 1.00E+00 |
| Metab:TG | -1.85E-03 | 6.06E-03 | 4.01E-01 | 4.01E-01 | 4.34E-01 | 5.10E-01 | 5.10E-01 | 1.00E+00 |
| Metab:FGlu | 6.63E-04 | 5.83E-03 | 3.63E-01 | 3.63E-01 | 4.25E-01 | 5.15E-01 | 5.15E-01 | 1.00E+00 |
| Metab:TgHDLcRatio | -1.25E-03 | 5.98E-03 | 3.64E-01 | 3.64E-01 | 4.04E-01 | 5.25E-01 | 5.25E-01 | 1.00E+00 |
| Pulm:FEF75 | -5.20E-03 | 6.78E-03 | 2.67E-01 | 2.67E-01 | 3.32E-01 | 5.65E-01 | 5.65E-01 | 1.00E+00 |
| BP:SysDiasRatio | -1.19E-04 | 5.82E-03 | 2.82E-01 | 2.82E-01 | 3.31E-01 | 5.65E-01 | 5.65E-01 | 1.00E+00 |
| Viro:sAgSeroClRate | 2.24E-02 | 4.54E-02 | 3.08E-01 | 3.08E-01 | 3.08E-01 | 5.79E-01 | 5.79E-01 | 1.00E+00 |
| Hepat:TBil | 7.00E-03 | 5.90E-03 | 2.58E-01 | 2.58E-01 | 2.95E-01 | 5.87E-01 | 5.87E-01 | 1.00E+00 |
| BP:DiasPulseRatio | -1.20E-04 | 5.82E-03 | 2.41E-01 | 2.41E-01 | 2.84E-01 | 5.94E-01 | 5.94E-01 | 1.00E+00 |
| Metab:HDLc | 3.20E-04 | 5.84E-03 | 2.40E-01 | 2.40E-01 | 2.80E-01 | 5.97E-01 | 5.97E-01 | 1.00E+00 |
| BP:Dias | 5.71E-03 | 5.81E-03 | 2.22E-01 | 2.22E-01 | 2.61E-01 | 6.09E-01 | 6.09E-01 | 1.00E+00 |
| Anthr:BAI | -1.80E-03 | 5.40E-03 | 1.83E-01 | 1.83E-01 | 2.50E-01 | 6.17E-01 | 6.17E-01 | 1.00E+00 |
| Viro:AtHCVAb | -2.79E-03 | 5.87E-03 | 1.96E-01 | 1.96E-01 | 2.26E-01 | 6.35E-01 | 6.35E-01 | 1.00E+00 |
| Pulm:Fev1FvcRatio | 2.40E-03 | 7.64E-03 | 2.30E-01 | 2.30E-01 | 2.25E-01 | 6.35E-01 | 6.35E-01 | 1.00E+00 |
| Habit:SMK_PkgYr | 9.66E-03 | 1.46E-02 | 1.75E-01 | 1.75E-01 | 1.88E-01 | 6.64E-01 | 6.64E-01 | 1.00E+00 |
| Viro:HBeAg | -2.47E-03 | 5.98E-03 | 1.62E-01 | 1.62E-01 | 1.80E-01 | 6.72E-01 | 6.72E-01 | 1.00E+00 |
| Habit:DRK_y | 6.14E-03 | 1.99E-02 | 1.10E-01 | 1.10E-01 | 1.42E-01 | 7.06E-01 | 7.06E-01 | 1.00E+00 |
| Pulm:EvFvcRatio | -2.63E-03 | 7.53E-03 | 1.29E-01 | 1.29E-01 | 1.30E-01 | 7.19E-01 | 7.19E-01 | 1.00E+00 |
| Pulm:Fef75HtRatio | -4.23E-03 | 6.98E-03 | 1.08E-01 | 1.08E-01 | 1.27E-01 | 7.22E-01 | 7.22E-01 | 1.00E+00 |
| BP:Pulse | 3.05E-03 | 5.64E-03 | 9.41E-02 | 9.41E-02 | 1.18E-01 | 7.32E-01 | 7.32E-01 | 1.00E+00 |
| Hepat:GgtAltRatio | 2.22E-03 | 6.27E-03 | 1.13E-01 | 1.13E-01 | 1.14E-01 | 7.35E-01 | 7.35E-01 | 1.00E+00 |
| Hepat:Alb | -8.83E-04 | 5.93E-03 | 9.22E-02 | 9.22E-02 | 1.04E-01 | 7.47E-01 | 7.47E-01 | 1.00E+00 |
| Metab:TCho | 1.96E-03 | 6.13E-03 | 9.85E-02 | 9.85E-02 | 1.04E-01 | 7.47E-01 | 7.47E-01 | 1.00E+00 |
| Pulm:MMF | -2.30E-03 | 6.75E-03 | 4.89E-02 | 4.89E-02 | 6.13E-02 | 8.04E-01 | 8.04E-01 | 1.00E+00 |
| BP:Sys | 6.33E-03 | 5.60E-03 | 4.52E-02 | 4.52E-02 | 5.74E-02 | 8.11E-01 | 8.11E-01 | 1.00E+00 |
| Metab:BMIAdjFGlu | 4.74E-03 | 5.98E-03 | 5.11E-02 | 5.11E-02 | 5.67E-02 | 8.12E-01 | 8.12E-01 | 1.00E+00 |
| Metab:TChoHDLcRatio | 1.36E-03 | 5.96E-03 | 3.51E-02 | 3.51E-02 | 3.93E-02 | 8.43E-01 | 8.43E-01 | 1.00E+00 |
| Viro:HBsAg | -2.04E-03 | 6.07E-03 | 1.93E-02 | 1.93E-02 | 2.08E-02 | 8.85E-01 | 8.85E-01 | 1.00E+00 |
| Metab:FGluHbA1cRatio | 3.36E-03 | 6.26E-03 | 1.12E-02 | 1.12E-02 | 1.13E-02 | 9.15E-01 | 9.15E-01 | 1.00E+00 |
| Pulm:Fiv1FvcRatio | 1.03E-04 | 8.16E-03 | 1.11E-02 | 1.11E-02 | 1.11E-02 | 9.16E-01 | 9.16E-01 | 1.00E+00 |
| Pulm:FEF50 | 1.86E-04 | 6.85E-03 | 5.02E-03 | 5.02E-03 | 6.12E-03 | 9.38E-01 | 9.38E-01 | 1.00E+00 |
| Metab:LDLc | 4.52E-04 | 6.22E-03 | 3.70E-03 | 3.70E-03 | 3.80E-03 | 9.51E-01 | 9.51E-01 | 1.00E+00 |
| Viro:AtHBsAb | -8.60E-04 | 5.57E-03 | 2.44E-03 | 2.44E-03 | 3.13E-03 | 9.55E-01 | 9.55E-01 | 1.00E+00 |
| Anthr:BF | -4.44E-03 | 5.12E-03 | 2.02E-04 | 2.02E-04 | 3.17E-04 | 9.86E-01 | 9.86E-01 | 1.00E+00 |
| Habit:Nut_DsYr | -9.09E-03 | 4.46E-02 | 6.08E-05 | 6.08E-05 | 6.50E-05 | 9.94E-01 | 9.94E-01 | 1.00E+00 |

| **Table S7.** The 84 binary traits (16 phenotype categories) are included in this study. | | | | |  |
| --- | --- | --- | --- | --- | --- |
| **No.** | **Category** | **Trait** | **Abbr.** | **Note** | |
| 1 | Alimentary canal diseases | Gastroesophageal reflux | GasRef |  | |
| 2 |  | Irritable bowel syndrome | IBS |  | |
| 3 |  | Peptic ulcer | PepUlc |  | |
| 4 | Arthritis | Arthritis | Arthr |  | |
| 5 |  | Adhesive capsulitis | AC |  | |
| 6 |  | Ankylosing spondylitis | AS |  | |
| 7 |  | Degenerative arthritis | DA |  | |
| 8 |  | Palindromic rheumatism | PR |  | |
| 9 |  | Psoriatic arthritis | PsA |  | |
| 10 |  | Rheumatoid arthritis | RA |  | |
| 11 | Orthopedic diseases | Osteoporosis | Osteo |  | |
| 12 | Cancers | Breast cancer | Brst | Female-only (for male, coded as missing) | |
| 13 |  | Cervical cancer | Cerv | Female-only (for male, coded as missing) | |
| 14 |  | Colorectal cancer | Colon |  | |
| 15 |  | Gastric cancer | Gast |  | |
| 16 |  | Liver cancer | Liv |  | |
| 17 |  | Lung cancer | Lung |  | |
| 18 |  | Nasopharyngeal cancer | Nasph |  | |
| 19 |  | Ovarian cancer | Ova | Female-only (for male, coded as missing) | |
| 20 |  | Uterine cancer | Uter | Female-only (for male, coded as missing) | |
| 21 |  | Prostate cancer | Prst | Male-only (for female, coded as missing) | |
| 22 |  | Other cancer | OtherCA |  | |
| 23 | Cardiovascular diseases | Arrhythmia | Arythm |  | |
| 24 |  | Congenital heart disease | CongHrt |  | |
| 25 |  | Coronary artery disease | CoroArt |  | |
| 26 |  | Cardiomyopathy | Crdmyo |  | |
| 27 |  | Valve heart disease | ValHrt |  | |
| 28 |  | Other heart disease | OtherHrt |  | |
| 29 | Gynecologic | Dysmenorrhea | Dys | Female-only (for male, coded as missing) | |
| 30 |  | Endometriosis | Endo | Female-only (for male, coded as missing) | |
| 31 |  | Ovarian cyst | OvaCys | Female-only (for male, coded as missing) | |
| 32 |  | Hormone drug usage for menopause | HorDrgMenps | Female-only (for male, coded as missing) | |
| 33 |  | Irregular menstruation | IrgMS | Female-only (for male, coded as missing) | |
| 34 |  | Myoma | Myo | Female-only (for male, coded as missing) | |
| 35 |  | Natural abortion | NatAbor | Had ever pregnant female-only (for nulliparous female and male, coded as missing) | |
| 36 | Habitual | Coffee consumption | Cof |  | |
| 37 |  | Tea consumption | Tea |  | |
| 38 |  | Snake consumption | Snak |  | |
| 39 |  | Drink | DRK |  | |
| 40 |  | Nut | NUT |  | |
| 41 |  | Smoke | SMK |  | |
| 42 |  | Sport | Spt |  | |
| 43 | Headache-induced symptom | Affect diary life when headache | AchDiary |  | |
| 44 |  | Nausea when headache | AchNau |  | |
| 45 |  | Photophobia when headache | AchePhot |  | |
| 46 | Hepatic symptom | Liver gall stone | LivGaStn |  | |
| 47 | Immune disease | Type 1 diabetes mellitus | tp1DM |  | |
| 48 | Metabolism disorders | Diabetes mellitus | DM |  | |
| 49 |  | Type 2 diabetes mellitus | tp2DM |  | |
| 50 |  | Gestational diabetes mellitus | tyGDM | Had ever pregnant female-only (for nulliparous female and male, coded as missing) | |
| 51 |  | Gout | Gout |  | |
| 52 |  | Hyperlipidemia | HypLip |  | |
| 53 |  | Hypertension | HypTens |  | |
| 54 |  | Stroke | Stroke |  | |
| 55 | Nephrotic symptoms | Kidney stone | KdnStn |  | |
| 56 |  | Renal failure | RenlFail |  | |
| 57 | Nervous system diseases | Dementia | Dmntia |  | |
| 58 |  | Epilepsy | Epilps |  | |
| 59 |  | Hemicrania | Hemcra |  | |
| 60 |  | Multiple sclerosis | MS |  | |
| 61 |  | Parkison’s disease | Prkins |  |  |
| 62 |  | Vertigo | Vrtig |  |  |
| 63 | Ophthalmic symptoms | Blind | Blnd |  |  |
| 64 |  | Color blind | ColBlnd |  |  |
| 65 |  | Cataract | Ctrc |  |  |
| 66 |  | Floaters | Flt |  |  |
| 67 |  | Glaucoma | Gluc |  |  |
| 68 |  | Retinal detachment | RntDe |  |  |
| 69 |  | Xerophthalmia | Xrph |  |  |
| 70 |  | Other eye disease | OtherEye |  |  |
| 71 | Psychiatric disorders | Alcoholism drug abuse | AlcoDrgAbuse |  |  |
| 72 |  | Depression | Deprs |  |  |
| 73 |  | Manic depression | ManDeprs |  |  |
| 74 |  | Obsessive compulsive disease | ObsComp |  |  |
| 75 |  | Postpartum depression | PostDeprs | Had ever pregnant female-only (for nulliparous female and male, coded as missing) |  |
| 76 |  | Schizophrenia | Schiz |  |  |
| 77 | Pulmonary syndromes | Asthma | Asthm |  |  |
| 78 |  | Emphysema bronchitis | EmphBrnch |  |  |
| 79 | Soreness | Articulus ache | Art |  |  |
| 80 |  | Back waist ache | BakWst |  |  |
| 81 |  | Headache | Head |  |  |
| 82 |  | Neckache | Nck |  |  |
| 83 |  | Sciatica | Sct |  |  |
| 84 |  | Other ache | OtherAch |  |  |
| Abbr., abbreviation of each trait used in this study | | | | |  |

| **Table S8.** Association results of rs75776403 and binary traits. | | | | | |
| --- | --- | --- | --- | --- | --- |
| **Traits** | **Beta** | **SE** | **PVal** | **PExtreme** | **PBonf** |
| Cardi:Crdmyo | -0.247598714 | 0.06534573 | 0.00015122 | 1.51E-04 | 0.012248824 |
| Nerve:Hemcra | -0.097753808 | 0.036830525 | 0.007950802 | 7.95E-03 | 0.644014999 |
| Metab:DM | -0.068535608 | 0.027632963 | 0.013130426 | 1.31E-02 | 1 |
| Gyn:Dys | 0.095803592 | 0.039207253 | 0.014544883 | 1.45E-02 | 1 |
| Nephr:KdnStn | -0.063496396 | 0.02599264 | 0.014571364 | 1.46E-02 | 1 |
| Cancer:Brst | 0.479423168 | 0.19772783 | 0.015322643 | 1.53E-02 | 1 |
| Metab:tp2DM | -0.066070706 | 0.028062505 | 0.018552023 | 1.86E-02 | 1 |
| Cancer:Ova | -1.991048828 | 0.849110169 | 0.019033963 | 1.90E-02 | 1 |
| Metab:Gout | -0.069791738 | 0.034990807 | 0.046089414 | 4.61E-02 | 1 |
| Pulm:Asthm | -0.065373585 | 0.03359458 | 0.051659985 | 5.17E-02 | 1 |
| Head:Vrtig | -0.046364326 | 0.024732183 | 0.060840115 | 6.08E-02 | 1 |
| Arthr:DA | -0.045726243 | 0.029965133 | 0.127014429 | 1.27E-01 | 1 |
| Gyn:NatAbor | 0.032011073 | 0.021507473 | 0.136653395 | 1.37E-01 | 1 |
| Ophth:RntDe | 0.074860666 | 0.052279005 | 0.152159531 | 1.52E-01 | 1 |
| Arthr:Arthr | -0.037472297 | 0.027138702 | 0.167349789 | 1.67E-01 | 1 |
| Habit:SMK | 0.022977594 | 0.017184271 | 0.181180402 | 1.81E-01 | 1 |
| Arthr:PsA | -0.692558225 | 0.518021711 | 0.181245828 | 1.81E-01 | 1 |
| Immun:tp1DM | -0.484599051 | 0.370230608 | 0.190564377 | 1.91E-01 | 1 |
| Gyn:HorDrgMenps | 0.061218758 | 0.050680189 | 0.227069353 | 2.27E-01 | 1 |
| Habit:DRK | 0.033556295 | 0.028680811 | 0.242004535 | 2.42E-01 | 1 |
| Bone:Osteo | 0.035061902 | 0.030776386 | 0.254600202 | 2.55E-01 | 1 |
| Metab:Stroke | -0.085442565 | 0.076241095 | 0.262420218 | 2.62E-01 | 1 |
| Habit:Cof | -0.026301144 | 0.023865588 | 0.270438601 | 2.70E-01 | 1 |
| Arthr:AS | -0.185512941 | 0.172320558 | 0.281678124 | 2.82E-01 | 1 |
| Sore:Head | -0.026826119 | 0.026023882 | 0.302621983 | 3.03E-01 | 1 |
| Cardi:CongHrt | 0.138311195 | 0.146354268 | 0.34463651 | 3.45E-01 | 1 |
| Cancer:Lung | 0.296237387 | 0.32135722 | 0.356616184 | 3.57E-01 | 1 |
| Arthr:PR | -0.32074329 | 0.362152375 | 0.375801636 | 3.76E-01 | 1 |
| Psych:PostDeprs | 0.102923659 | 0.121836472 | 0.398239897 | 3.98E-01 | 1 |
| Psych:Schiz | 0.130202316 | 0.154230107 | 0.398553091 | 3.99E-01 | 1 |
| Sore:BakWst | -0.017255299 | 0.02127007 | 0.417223359 | 4.17E-01 | 1 |
| Cancer:Prst | 0.388901858 | 0.486868195 | 0.424416464 | 4.24E-01 | 1 |
| Cancer:Cerv | -0.285972998 | 0.362993129 | 0.430802402 | 4.31E-01 | 1 |
| Cardi:Arythm | 0.022374692 | 0.028898235 | 0.438778186 | 4.39E-01 | 1 |
| Psych:Deprs | 0.024412834 | 0.032651462 | 0.454653404 | 4.55E-01 | 1 |
| Metab:tyGDM | 0.112147571 | 0.15076195 | 0.456954008 | 4.57E-01 | 1 |
| Sore:Other | 0.031104084 | 0.041952167 | 0.458440141 | 4.58E-01 | 1 |
| Cancer:Nasph | 0.69788033 | 0.969441176 | 0.471599508 | 4.72E-01 | 1 |
| Sore:Nck | 0.014851833 | 0.021040807 | 0.480276115 | 4.80E-01 | 1 |
| Cardi:CoroArt | -0.037601478 | 0.054560096 | 0.490712874 | 4.91E-01 | 1 |
| Ophth:Flt | -0.012801795 | 0.018681373 | 0.493173192 | 4.93E-01 | 1 |
| Hepat:LivGaStn | 0.019220209 | 0.029300108 | 0.511838671 | 5.12E-01 | 1 |
| Cardi:Other | -0.097953589 | 0.155365156 | 0.528384942 | 5.28E-01 | 1 |
| Cancer:Other | 0.113196501 | 0.180157275 | 0.529794034 | 5.30E-01 | 1 |
| Sore:Arthr | 0.014477652 | 0.023193679 | 0.532491788 | 5.32E-01 | 1 |
| Cancer:Liv | -0.32882931 | 0.533511673 | 0.537664299 | 5.38E-01 | 1 |
| Alimn:IBS | 0.022886075 | 0.03983848 | 0.565648714 | 5.66E-01 | 1 |
| Psych:ObsComp | 0.123778335 | 0.217559229 | 0.569396277 | 5.69E-01 | 1 |
| Ophth:Other | 0.018482196 | 0.033408056 | 0.580108862 | 5.80E-01 | 1 |
| Metab:HypTens | -0.010590488 | 0.019196554 | 0.581162855 | 5.81E-01 | 1 |
| Ophth:Blnd | -0.079329176 | 0.146573301 | 0.588352313 | 5.88E-01 | 1 |
| Ophth:Xrph | 0.010399229 | 0.019366531 | 0.591288948 | 5.91E-01 | 1 |
| Gyn:Endo | 0.015027345 | 0.029426031 | 0.609573732 | 6.10E-01 | 1 |
| Gyn:Myo | 0.009112338 | 0.018547608 | 0.623218147 | 6.23E-01 | 1 |
| Habit:Spt | -0.006464564 | 0.013580001 | 0.634049 | 6.34E-01 | 1 |
| Ophth:Ctrc | 0.010179966 | 0.022271343 | 0.647607726 | 6.48E-01 | 1 |
| Nerve:Dmntia | -0.127633813 | 0.28811297 | 0.657766319 | 6.58E-01 | 1 |
| Metab:HypLip | 0.009600893 | 0.023067101 | 0.67725206 | 6.77E-01 | 1 |
| Habit:Snak | -0.008106227 | 0.025902344 | 0.754316054 | 7.54E-01 | 1 |
| Nerve:Prkins | -0.048156235 | 0.162051736 | 0.766339926 | 7.66E-01 | 1 |
| Pulm:EmphBrnch | -0.016487565 | 0.057822573 | 0.775536408 | 7.76E-01 | 1 |
| Sore:Sct | 0.011228334 | 0.04074391 | 0.782868273 | 7.83E-01 | 1 |
| Habit:Tea | 0.006439166 | 0.027445009 | 0.814503174 | 8.15E-01 | 1 |
| Gyn:IrgMS | 0.00430103 | 0.020599641 | 0.834610995 | 8.35E-01 | 1 |
| Cardi:ValHrt | 0.00602843 | 0.030359872 | 0.842602472 | 8.43E-01 | 1 |
| Alimn:GasRef | -0.003253143 | 0.017788874 | 0.854895977 | 8.55E-01 | 1 |
| Gyn:OvaCys | -0.005843519 | 0.032562459 | 0.857579888 | 8.58E-01 | 1 |
| Arthr:AC | -0.108110067 | 0.622195647 | 0.862057427 | 8.62E-01 | 1 |
| Nephr:RenlFail | -0.025595718 | 0.171003194 | 0.881017273 | 8.81E-01 | 1 |
| Nerve:Epilps | -0.015580028 | 0.11054543 | 0.887919091 | 8.88E-01 | 1 |
| Ophth:ColBlnd | -0.013314017 | 0.099744189 | 0.893812489 | 8.94E-01 | 1 |
| Habit:NUT | -0.003817064 | 0.028949998 | 0.895102757 | 8.95E-01 | 1 |
| Psych:ManDeprs | -0.00753174 | 0.076314335 | 0.921381518 | 9.21E-01 | 1 |
| Arthr:RA | 0.006426769 | 0.068791678 | 0.925567013 | 9.26E-01 | 1 |
| Alimn:PepUlc | 0.001480579 | 0.017503333 | 0.932588631 | 9.33E-01 | 1 |
| Ophth:Gluc | -0.004190109 | 0.05224515 | 0.936077465 | 9.36E-01 | 1 |
| Psych:AlcoDrgAbuse | -0.026031255 | 0.335970574 | 0.938241096 | 9.38E-01 | 1 |
| Cancer:Uter | 0.038287844 | 0.523283478 | 0.941672067 | 9.42E-01 | 1 |
| Cancer:Gast | -0.06307855 | 1.139660898 | 0.955860804 | 9.56E-01 | 1 |
| Cancer:Colon | -0.010729708 | 0.309058231 | 0.97230506 | 9.72E-01 | 1 |
| Nerve:MS | -0.003964866 | 0.43895914 | 0.992793266 | 9.93E-01 | 1 |

| **Table S9.** The 19 ordered traits (4 phenotype categories) are included in this study. | | | | | |
| --- | --- | --- | --- | --- | --- |
| **No.** | **Category** | **Trait** | **Abbr.** | **Type** | **Note** |
| 1 | Soreness | Articulus ache frequency | ArtFrq | 0/1/2 | 0:No, 1:Seldom, 2:Often |
| 2 |  | Neckache frequency | NckFrq | 0/1/2 | 0:No, 1:Seldom, 2:Often |
| 3 |  | Back waist ache frequency | BakWstFrq | 0/1/2 | 0:No, 1:Seldom, 2:Often |
| 4 |  | Sciatica frequency | SctFrq | 0/1/2 | 0:No, 1:Seldom, 2:Often |
| 5 |  | Headache frequency | HeadFrq | 0/1/2 | 0:No, 1:Seldom, 2:Often |
| 6 |  | Dysmenorrhea frequency | DsmFrq | 0/1/2 | 0:No, 1:Seldom, 2:Often; Female-only (for male, coded as missing) |
| 7 |  | Headache severity | HeadSvr | 1/2/3 | 1:Light,2:Medium,3:Severe |
| 8 | Ophthalmologic | Cataract eye number | CtrcN | 0/1/2 | 0:No, 1:Single eye, 2:Both eyes; Single eye: only left eye or right eye had disease by questionnaire; Both eyes: both ‌left eye and right eye had disease by questionnaire |
| 9 |  | Glaucoma eye number | GlucN | 0/1/2 | 0:No, 1:Single eye, 2:Both eyes |
| 10 |  | Xerophthalmia eye number | XrphN | 0/1/2 | 0:No, 1:Single eye, 2:Both eyes |
| 11 |  | Retinal detachment eye number | RntDeN | 0/1/2 | 0:No, 1:Single eye, 2:Both eyes |
| 12 |  | Floaters eye number | FltN | 0/1/2 | 0:No, 1:Single eye, 2:Both eyes |
| 13 |  | Blind eye number | BlndN | 0/1/2 | 0:No, 1:Single eye, 2:Both eyes |
| 14 |  | Color blind eye number | ColBlndN | 0/1/2 | 0:No, 1:Single eye, 2:Both eyes |
| 15 | Habitual | Snake consumption frequency | SNKFrq | 1/2/3/4/5 | 1:Less than once per month, 2:One to three times per month, 3:One to three times per week, 4:Four to six times per week, 5: Almost everyday |
| 16 | Psychiatric | Nervous | Nerv | 0/1/2/3 | 0:No, 1:Few days, 2: More than half of week, 3:Almost everyday |
| 17 |  | Anxiety | Anxt | 0/1/2/3 | 0:No, 1:Few days, 2: More than half of week, 3:Almost everyday |
| 18 |  | Down | Dwn | 0/1/2/3 | 0:No, 1:Few days, 2: More than half of week, 3:Almost everyday |
| 19 |  | Depression | Dprs | 0/1/2/3 | 0:No, 1:Few days, 2: More than half of week, 3:Almost everyday |
| Abbr., abbreviation of each trait used in this study. | | | | | |

| **Table S10.** Association results of rs75776403 and ordinary traits. | | | | | |
| --- | --- | --- | --- | --- | --- |
| **Traits** | **Beta** | **SE** | **PVal** | **PExtreme** | **PBonf** |
| Habit:SNKFrq | 9.55E-02 | 3.79E-02 | 1.17E-02 | 1.17E-02 | 1.87E-01 |
| Ophth:RntDeN | 8.99E-02 | 4.22E-02 | 3.33E-02 | 3.33E-02 | 5.32E-01 |
| Sore:DsmFrq | 7.09E-02 | 5.72E-02 | 2.16E-01 | 2.16E-01 | 1.00E+00 |
| Psych:Nerv | 3.02E-02 | 2.50E-02 | 2.27E-01 | 2.27E-01 | 1.00E+00 |
| Sore:HeadSvr | 5.88E-02 | 5.09E-02 | 2.48E-01 | 2.48E-01 | 1.00E+00 |
| Psych:Dwn | 3.47E-02 | 3.16E-02 | 2.73E-01 | 2.73E-01 | 1.00E+00 |
| Sore:HeadFrq | -2.77E-02 | 2.54E-02 | 2.75E-01 | 2.75E-01 | 1.00E+00 |
| Ophth:XrphN | 1.79E-02 | 1.94E-02 | 3.57E-01 | 3.57E-01 | 1.00E+00 |
| Psych:Anxt | 4.46E-02 | 4.97E-02 | 3.69E-01 | 3.69E-01 | 1.00E+00 |
| Sore:NckFrq | 1.66E-02 | 2.02E-02 | 4.11E-01 | 4.11E-01 | 1.00E+00 |
| Sore:BakWstFrq | -1.65E-02 | 2.11E-02 | 4.34E-01 | 4.34E-01 | 1.00E+00 |
| Psych:Dprs | 2.37E-02 | 3.26E-02 | 4.67E-01 | 4.67E-01 | 1.00E+00 |
| Sore:ArthrFrq | 1.45E-02 | 2.25E-02 | 5.21E-01 | 5.21E-01 | 1.00E+00 |
| Sore:SctFrq | 1.10E-02 | 3.03E-02 | 7.16E-01 | 7.16E-01 | 1.00E+00 |
| Ophth:FltN | -6.65E-03 | 1.87E-02 | 7.22E-01 | 7.22E-01 | 1.00E+00 |
| Ophth:CtrcN | -1.96E-04 | 2.31E-02 | 9.93E-01 | 9.93E-01 | 1.00E+00 |

| **Table S11.** Phenome-wide association study for *CLEC18A* rs75776403 in UK Biobank (UKB) and BioBank Japan (BBJ). | | | | | | |  |
| --- | --- | --- | --- | --- | --- | --- | --- |
| **Trait** | **Type** | **Sample size** | ***β*** | **Std. Err.** | ***P*** | **Fdr** |  |
| **UK Biobank (Trait No.: 2173)** |  |  |  |  |  |  |  |
| Sitting height | Quant. | 435693 | -0.0135 | 0.0018 | 2.86×10^-13^ | 6.21×10^-10^ |  |
| Forced expiratory volume in 1-second (FEV1) | Quant. | 398413 | -0.0128 | 0.0019 | 1.51×10^-11^ | 1.35×10^-8^ |  |
| Forced vital capacity (FVC), Best measure | Quant. | 328127 | -0.0135 | 0.0020 | 1.87×10^-11^ | 1.35×10^-8^ |  |
| Forced expiratory volume in 1-second (FEV1), Best measure | Quant. | 328127 | -0.0136 | 0.0021 | 6.08×10^-11^ | 3.16×10^-8^ |  |
| Forced vital capacity (FVC) | Quant. | 398413 | -0.0120 | 0.0018 | 7.27×10^-11^ | 3.16×10^-8^ |  |
| Forced expiratory volume in 1-second (FEV1), predicted percentage | Quant. | 141284 | -0.0243 | 0.0043 | 2.04×10^-8^ | 7.09×10^-6^ |  |
| Smoking status: Never | Binary | 435218 | -0.0068 | 0.0012 | 2.28×10^-8^ | 7.09×10^-6^ |  |
| Past tobacco smoking | Ordinal | 401323 | -0.0167 | 0.0032 | 1.71×10^-7^ | 4.65×10^-5^ |  |
| Eosinophil count | Quant. | 423262 | 0.0110 | 0.0025 | 1.01×10^-5^ | 2.43×10^-3^ |  |
| Hand grip strength (right) | Quant. | 435250 | -0.0074 | 0.0017 | 1.35×10^-5^ | 2.93×10^-3^ |  |
| Forced expiratory volume in 1-second (FEV1), predicted | Quant. | 141284 | -0.0078 | 0.0018 | 1.63×10^-5^ | 3.22×10^-3^ |  |
| Ever smoked | Binary | 435267 | 0.0049 | 0.0012 | 4.10×10^-5^ | 7.41×10^-3^ |  |
| Standing height | Quant. | 436077 | -0.0070 | 0.0017 | 4.47×10^-5^ | 7.46×10^-3^ |  |
| Lymphocyte count | Quant. | 423262 | 0.0097 | 0.0025 | 9.36×10^-5^ | 1.45×10^-2^ |  |
| Age when periods started (menarche) | Ordinal | 230264 | -0.0095 | 0.0025 | 1.50×10^-4^ | 2.17×10^-2^ |  |
| Smoking status: Previous | Binary | 435218 | 0.0044 | 0.0012 | 1.77×10^-4^ | 2.40×10^-2^ |  |
| Beef intake | Ordinal | 435293 | 0.0074 | 0.0020 | 2.55×10^-4^ | 3.22×10^-2^ |  |
| Place of birth in UK - north co-ordinate | Quant. | 406521 | -0.0071 | 0.0019 | 2.79×10^-4^ | 3.22×10^-2^ |  |
| Wheeze or whistling in the chest in last year | Binary | 428654 | 0.0037 | 0.0010 | 2.82×10^-4^ | 3.22×10^-2^ |  |
| Number of depression episodes | Ordinal | 54929 | -0.0218 | 0.0061 | 3.46×10^-4^ | 3.75×10^-2^ |  |
| **BioBank Japan (Trait No.:229)** |  |  |  |  |  |  |  |
| Height | Quant. | 165056 | -0.015 | 0.0022 | 6.6×10^-11^ | 1.51×10^-8^ |  |
| Body weight | Quant. | 165419 | -0.013 | 0.0030 | 2.2×10^-5^ | 5.02×10^-3^ |  |
| Serum creatinine | Quant. | 150266 | -0.013 | 0.0031 | 2.7×10^-5^ | 6.13×10^-3^ |  |
| Std. Err., standard error; Fdr, local false discovery rate. Traits with a Fdr<0.05 were listed. | | | | | | | |

| **Table S12.** Significantly enriched pathways (and/or gene sets) were identified by gene-set enrichment analysis (GSEA). | | |
| --- | --- | --- |
| **Source** | **Dir.** | **Pathways** |
| mRNA | Pos. | (1) KEGG BIOSYNTHESIS OF UNSATURATED FATTY ACIDS; (2) KEGG DNA REPLICATION; (3) KEGG OOCYTE MEIOSIS; (4) KEGG PROGESTERONE MEDIATED OOCYTE MATURATION; (5) KEGG SPLICEOSOME; (6) KEGG CELL CYCLE; (7) REACTOME RESOLUTION OF SISTER CHROMATID COHESION; (8) REACTOME MITOTIC PROMETAPHASE; (9) REACTOME RHO GTPASES ACTIVATE FORMINS; (10) REACTOME SEPARATION OF SISTER CHROMATIDS; (11) REACTOME MITOTIC SPINDLE CHECKPOINT; (12) REACTOME DNA REPLICATION; (13) REACTOME REGULATION OF TP53 ACTIVITY THROUGH PHOSPHORYLATION; (14) REACTOME RECRUITMENT OF NUMA TO MITOTIC CENTROSOMES; (15) REACTOME MITOTIC METAPHASE AND ANAPHASE; (16) REACTOME S PHASE; (17) REACTOME MITOTIC G2-G2/M PHASES; (18) REACTOME CELL CYCLE; (19) REACTOME CELL CYCLE CHECKPOINTS; (20) REACTOME CELL CYCLE MITOTIC; (21) REACTOME PROCESSING OF CAPPED INTRON CONTAINING PRE MRNA; (22) REACTOME M PHASE; (23) REACTOME TRANSMISSION ACROSS CHEMICAL SYNAPSES; (24) REACTOME ORGANELLE BIOGENESIS AND MAINTENANCE; (25) REACTOME DNA REPAIR; (26) REACTOME ACTIVATION OF ATR IN RESPONSE TO REPLICATION STRESS; (27) REACTOME ALPHA LINOLENIC OMEGA3 AND LINOLEIC OMEGA6 ACID METABOLISM; (28) REACTOME SEMA3A PLEXIN REPULSION SIGNALING BY INHIBITING INTEGRIN ADHESION; (29) REACTOME DNA REPLICATION PRE INITIATION; (30) REACTOME TRANSPORT OF MATURE TRANSCRIPT TO CYTOPLASM; (31) REACTOME SWITCHING OF ORIGINS TO A POST REPLICATIVE STATE; (32) REACTOME MITOTIC G1 PHASE AND G1 S TRANSITION; (33) REACTOME CILIUM ASSEMBLY; (34) REACTOME NEUROTRANSMITTER RECEPTORS AND POSTSYNAPTIC SIGNAL TRANSMISSION; (35) REACTOME TRANSCRIPTIONAL REGULATION BY TP53; (36) REACTOME GLUCAGON TYPE LIGAND RECEPTORS; (37) REACTOME RECYCLING PATHWAY OF L1; (38) REACTOME ACTIVATION OF THE PRE REPLICATIVE COMPLEX; (39) REACTOME AGGREPHAGY; (40) REACTOME CARBOXYTERMINAL POST TRANSLATIONAL MODIFICATIONS OF TUBULIN; (41) REACTOME OPIOID SIGNALLING; (42) REACTOME REGULATION OF TP53 ACTIVITY; (43) REACTOME G2 M CHECKPOINTS; (44) REACTOME MRNA SPLICING; (45) REACTOME NEURONAL SYSTEM; (46) REACTOME CRMPS IN SEMA3A SIGNALING; (47) REACTOME LINOLEIC ACID LA METABOLISM; (48) REACTOME ORC1 REMOVAL FROM CHROMATIN; (49) REACTOME AURKA ACTIVATION BY TPX2; (50) REACTOME TRANSLOCATION OF SLC2A4 GLUT4 TO THE PLASMA MEMBRANE; (51) REACTOME REGULATION OF PLK1 ACTIVITY AT G2 M TRANSITION; (52) REACTOME ASSEMBLY OF THE PRE REPLICATIVE COMPLEX; (53) REACTOME RECRUITMENT OF MITOTIC CENTROSOME PROTEINS AND COMPLEXES; (54) REACTOME ACTIVATION OF NMDA RECEPTORS AND POSTSYNAPTIC EVENTS; (55) REACTOME TRANSPORT OF CONNEXONS TO THE PLASMA MEMBRANE; (56) REACTOME DNA STRAND ELONGATION; (57) REACTOME GLUCAGON LIKE PEPTIDE 1 GLP1 REGULATES INSULIN SECRETION; (58) REACTOME TRANSCRIPTIONAL REGULATION BY E2F6; (59) REACTOME EXTENSION OF TELOMERES; (60) REACTOME ASSEMBLY AND CELL SURFACE PRESENTATION OF NMDA RECEPTORS; (61) REACTOME ANCHORING OF THE BASAL BODY TO THE PLASMA MEMBRANE; (62) REACTOME RHO GTPASE EFFECTORS; (63) REACTOME G BETA GAMMA SIGNALLING THROUGH CDC42; (64) REACTOME PROTEIN FOLDING; (65) REACTOME ACTIVATION OF AMPK DOWNSTREAM OF NMDARS; (66) REACTOME KINESINS; (67) REACTOME NUCLEAR ENVELOPE NE REASSEMBLY; (68) REACTOME HOMOLOGOUS DNA PAIRING AND STRAND EXCHANGE; (69) REACTOME HDR THROUGH SINGLE STRAND ANNEALING SSA; (70) REACTOME HEDGEHOG OFF STATE; (71) REACTOME SUMOYLATION OF DNA REPLICATION PROTEINS; (72) REACTOME PRESYNAPTIC FUNCTION OF KAINATE RECEPTORS; (73) REACTOME SUMOYLATION OF DNA DAMAGE RESPONSE AND REPAIR PROTEINS; (74) REACTOME RHO GTPASES ACTIVATE IQGAPS; (75) REACTOME SUPPRESSION OF PHAGOSOMAL MATURATION; (76) REACTOME TRANSLESION SYNTHESIS BY POLH; (77) REACTOME GLYCOSPHINGOLIPID METABOLISM; (78) REACTOME SUMOYLATION OF RNA BINDING PROTEINS; (79) REACTOME ACTIVATION OF KAINATE RECEPTORS UPON GLUTAMATE BINDING; (80) REACTOME GLYCOLYSIS; (81) REACTOME COPI DEPENDENT GOLGI TO ER RETROGRADE TRAFFIC; (82) REACTOME PROSTACYCLIN SIGNALLING THROUGH PROSTACYCLIN RECEPTOR; (83) REACTOME NUCLEAR ENVELOPE BREAKDOWN; (84) REACTOME TRNA PROCESSING; (85) REACTOME HOST INTERACTIONS OF HIV FACTORS; (86) REACTOME HDR THROUGH HOMOLOGOUS RECOMBINATION HRR; (87) REACTOME POST CHAPERONIN TUBULIN FOLDING PATHWAY; (88) REACTOME RESPONSE OF MTB TO PHAGOCYTOSIS; (89) REACTOME DNA DOUBLE STRAND BREAK REPAIR; (90) REACTOME ADRENALINE NORADRENALINE INHIBITS INSULIN SECRETION; (91) REACTOME TRANSPORT OF MATURE MRNAS DERIVED FROM INTRONLESS TRANSCRIPTS; (92) REACTOME INTEGRATION OF ENERGY METABOLISM; (93) GOBP MITOTIC SISTER CHROMATID SEGREGATION; (94) GOBP SISTER CHROMATID SEGREGATION; (95) GOBP DNA REPLICATION; (96) GOBP DNA DEPENDENT DNA REPLICATION; (97) GOBP PROTEIN LOCALIZATION TO CHROMOSOME; (98) GOBP NUCLEAR CHROMOSOME SEGREGATION; (99) GOBP REGULATION OF DNA REPLICATION; (100) GOBP CHROMOSOME SEGREGATION; (101) GOBP CELL CYCLE DNA REPLICATION; (102) GOBP NEURAL NUCLEUS DEVELOPMENT; (103) GOBP PROTEIN LOCALIZATION TO SYNAPSE; (104) GOBP RECOMBINATIONAL REPAIR; (105) GOBP REGULATION OF MICROTUBULE POLYMERIZATION OR DEPOLYMERIZATION; (106) GOBP MITOTIC SPINDLE ORGANIZATION; (107) GOBP MICROTUBULE POLYMERIZATION OR DEPOLYMERIZATION; (108) GOBP MITOTIC NUCLEAR DIVISION; (109) GOBP MRNA EXPORT FROM NUCLEUS; (110) GOBP RNA EXPORT FROM NUCLEUS; (111) GOBP MICROTUBULE ORGANIZING CENTER ORGANIZATION; (112) GOBP REGULATION OF MICROTUBULE CYTOSKELETON ORGANIZATION; (113) GOBP REGULATION OF MRNA SPLICING VIA SPLICEOSOME; (114) GOBP SPINDLE ORGANIZATION; (115) GOBP DNA RECOMBINATION; (116) GOBP ORGANELLE FISSION; (117) GOBP MICROTUBULE CYTOSKELETON ORGANIZATION; (118) GOBP CHROMOSOME ORGANIZATION; (119) GOBP REGULATION OF CHROMOSOME ORGANIZATION; (120) GOBP DNA REPAIR; (121) GOBP RNA SPLICING VIA TRANSESTERIFICATION REACTIONS; (122) GOBP COVALENT CHROMATIN MODIFICATION; (123) GOBP DOUBLE STRAND BREAK REPAIR; (124) GOBP MICROTUBULE BASED PROCESS; (125) GOBP DNA METABOLIC PROCESS; (126) GOBP MITOTIC CELL CYCLE; (127) GOBP PROTEIN CONTAINING COMPLEX LOCALIZATION; (128) GOBP CELL DIVISION; (129) GOBP CELL CYCLE PROCESS; (130) GOBP RNA SPLICING; (131) GOBP MACROMOLECULE METHYLATION; (132) GOBP CELL CYCLE; (133) GOBP METHYLATION; (134) GOBP REGULATION OF DNA METABOLIC PROCESS; (135) GOBP MRNA PROCESSING; (136) GOBP CELL CYCLE PHASE TRANSITION; (137) GOBP PEPTIDYL LYSINE MODIFICATION; (138) GOBP CHROMATIN ORGANIZATION; (139) GOBP REGULATION OF CELL CYCLE PROCESS; (140) GOBP POSITIVE REGULATION OF CELL CYCLE; (141) GOBP CELLULAR RESPONSE TO DNA DAMAGE STIMULUS; (142) GOBP CYTOSKELETON ORGANIZATION; (143) GOBP REGULATION OF CELL CYCLE; (144) GOBP REGULATION OF ORGANELLE ORGANIZATION; (145) GOBP PYRIMIDINE NUCLEOBASE METABOLIC PROCESS; (146) GOBP ALPHA LINOLENIC ACID METABOLIC PROCESS; (147) GOBP SISTER CHROMATID COHESION; (148) GOBP REGULATION OF MICROTUBULE POLYMERIZATION; (149) GOBP REGULATION OF CHROMOSOME SEGREGATION; (150) GOBP METAPHASE ANAPHASE TRANSITION OF CELL CYCLE; (151) GOBP MICROTUBULE POLYMERIZATION; (152) GOBP MEIOTIC CHROMOSOME SEGREGATION; (153) GOBP CHROMOSOME LOCALIZATION; (154) GOBP NEGATIVE REGULATION OF CHROMOSOME ORGANIZATION; (155) GOBP ADULT BEHAVIOR; (156) GOBP MICROTUBULE CYTOSKELETON ORGANIZATION INVOLVED IN MITOSIS; (157) GOBP PEPTIDYL LYSINE METHYLATION; (158) GOBP REGULATION OF RNA SPLICING; (159) GOBP PROTEIN METHYLATION; (160) GOBP REGULATION OF MITOTIC CELL CYCLE; (161) GOBP SYNAPTIC VESICLE CYTOSKELETAL TRANSPORT; (162) GOBP CHROMOSOME SEPARATION; (163) GOBP CILIARY BASAL BODY PLASMA MEMBRANE DOCKING; (164) GOBP REGULATION OF MRNA PROCESSING; (165) GOBP MRNA TRANSPORT; (166) GOBP CENTRAL NERVOUS SYSTEM NEURON DIFFERENTIATION; (167) GOBP PROTEIN ACYLATION; (168) GOBP REGULATION OF CELL CYCLE G2 M PHASE TRANSITION; (169) GOBP REGULATION OF MICROTUBULE BASED PROCESS; (170) GOBP MEIOTIC CELL CYCLE; (171) GOBP SUPRAMOLECULAR FIBER ORGANIZATION; (172) GOBP REGULATION OF DNA DEPENDENT DNA REPLICATION; (173) GOBP DNA DEPENDENT DNA REPLICATION MAINTENANCE OF FIDELITY; (174) GOBP DNA UNWINDING INVOLVED IN DNA REPLICATION; (175) GOBP POSITIVE REGULATION OF CELL CELL ADHESION MEDIATED BY CADHERIN; (176) GOBP CHROMOSOME ORGANIZATION INVOLVED IN MEIOTIC CELL CYCLE; (177) GOBP NEURON FATE COMMITMENT; (178) GOBP POSITIVE REGULATION OF MEMBRANE PERMEABILITY; (179) GOBP METAPHASE PLATE CONGRESSION; (180) GOBP REGULATION OF MITOTIC NUCLEAR DIVISION; (181) GOBP DNA GEOMETRIC CHANGE; (182) GOBP MEIOTIC CELL CYCLE PROCESS; (183) GOBP RNA LOCALIZATION; (184) GOBP NEGATIVE REGULATION OF ORGANELLE ORGANIZATION; (185) GOBP ORGANELLE LOCALIZATION; (186) GOBP RESPONSE TO HYDROXYUREA; (187) GOBP ANGIOTENSIN ACTIVATED SIGNALING PATHWAY; (188) GOBP NATURAL KILLER CELL DEGRANULATION; (189) GOBP CEREBELLAR GRANULAR LAYER MORPHOGENESIS; (190) GOBP NEGATIVE REGULATION OF ADENYLATE CYCLASE ACTIVATING G PROTEIN COUPLED RECEPTOR SIGNALING PATHWAY; (191) GOBP CELL CYCLE G2 M PHASE TRANSITION; (192) GOBP POSITIVE REGULATION OF GASTRULATION; (193) GOBP REGULATION OF CHROMOSOME SEPARATION; (194) GOBP RNA PROCESSING; (195) GOBP REGULATION OF CELL CELL ADHESION MEDIATED BY CADHERIN; (196) GOBP HISTONE METHYLATION; (197) GOBP CELLULAR RESPONSE TO UV B; (198) GOBP POSTREPLICATION REPAIR; (199) GOBP INTERSTRAND CROSS LINK REPAIR; (200) GOBP HOMOLOGOUS RECOMBINATION; (201) GOBP ALTERNATIVE MRNA SPLICING VIA SPLICEOSOME; (202) GOBP CEREBELLAR CORTEX DEVELOPMENT; (203) GOBP NEGATIVE REGULATION OF MRNA PROCESSING; (204) GOBP DORSAL SPINAL CORD DEVELOPMENT; (205) GOBP CEREBELLAR GRANULAR LAYER DEVELOPMENT; (206) GOBP CORTICOSTEROID RECEPTOR SIGNALING PATHWAY; (207) GOBP MICROTUBULE SEVERING; (208) GOBP CYTOKINESIS; (209) GOBP REGULATION OF CELL CYCLE PHASE TRANSITION; (210) GOBP HISTONE DEACETYLATION; (211) GOBP CENTRAL NERVOUS SYSTEM NEURON DEVELOPMENT; (212) GOBP GLUTATHIONE CATABOLIC PROCESS; (213) GOBP MEIOSIS I CELL CYCLE PROCESS; (214) GOBP PEPTIDYL LYSINE ACETYLATION; (215) GOBP ESTABLISHMENT OF RNA LOCALIZATION; (216) GOBP REGULATION OF PROTEIN POLYMERIZATION; (217) GOBP PROTEIN POLYMERIZATION; (218) GOBP DNA CONFORMATION CHANGE; (219) GOBP TELOMERE MAINTENANCE VIA TELOMERE LENGTHENING; (220) GOBP PYRIMIDINE CONTAINING COMPOUND METABOLIC PROCESS; (221) GOBP REGULATION OF DOUBLE STRAND BREAK REPAIR; (222) GOBP RESPONSE TO UV B; (223) GOBP NEGATIVE REGULATION OF METAPHASE ANAPHASE TRANSITION OF CELL CYCLE; (224) GOBP REGULATION OF MITOTIC SISTER CHROMATID SEGREGATION; (225) GOBP REGULATION OF PROTEIN DEACETYLATION; (226) GOBP G0 TO G1 TRANSITION; (227) GOBP PROTEIN ACETYLATION; (228) GOBP ETHANOLAMINE CONTAINING COMPOUND METABOLIC PROCESS; (229) GOBP POSITIVE REGULATION OF STEROID METABOLIC PROCESS; (230) GOBP CELLULAR PROTEIN CONTAINING COMPLEX ASSEMBLY; (231) GOBP EPITHELIAL CELL MATURATION; (232) GOBP REGULATION OF CHONDROCYTE DEVELOPMENT; (233) GOBP NEGATIVE REGULATION OF ACUTE INFLAMMATORY RESPONSE TO ANTIGENIC STIMULUS; (234) GOBP RESPONSE TO ANTIBIOTIC; (235) GOBP NEUROTRANSMITTER SECRETION; (236) GOBP POSITIVE REGULATION OF CHROMOSOME ORGANIZATION; (237) GOBP UBIQUINONE METABOLIC PROCESS; (238) GOBP PYRIMIDINE CONTAINING COMPOUND BIOSYNTHETIC PROCESS; (239) GOBP REGULATION OF NUCLEAR DIVISION; (240) GOBP POSITIVE REGULATION OF CELL CYCLE PROCESS; (241) GOBP SPINAL CORD DEVELOPMENT; (242) GOBP NEGATIVE REGULATION OF RNA SPLICING; (243) GOBP CYTOPLASMIC MICROTUBULE ORGANIZATION; (244) GOBP REGULATION OF CELLULAR RESPONSE TO HEAT; (245) GOBP NEURON DEVELOPMENT; (246) GOBP REGULATION OF CELL CYCLE CHECKPOINT; (247) GOBP DEOXYRIBONUCLEOTIDE BIOSYNTHETIC PROCESS; (248) GOBP NEGATIVE REGULATION OF EXCITATORY POSTSYNAPTIC POTENTIAL; (249) GOBP POSITIVE REGULATION OF HISTONE METHYLATION; (250) GOBP OLIGODENDROCYTE DIFFERENTIATION; (251) GOBP NEGATIVE REGULATION OF CELL CYCLE PROCESS; (252) GOBP CARBOHYDRATE METABOLIC PROCESS; (253) GOBP PROTEIN CONTAINING COMPLEX SUBUNIT ORGANIZATION; (254) GOBP POSTSYNAPTIC SPECIALIZATION ASSEMBLY; (255) GOBP TRANSLESION SYNTHESIS; (256) GOBP ANTEROGRADE AXONAL TRANSPORT; (257) GOBP REGULATION OF DOUBLE STRAND BREAK REPAIR VIA HOMOLOGOUS RECOMBINATION; (258) GOBP REGULATION OF NEUROTRANSMITTER LEVELS; (259) GOBP LINOLEIC ACID METABOLIC PROCESS; (260) GOBP ERROR FREE TRANSLESION SYNTHESIS; (261) GOBP PEPTIDYL L CYSTEINE S PALMITOYLATION; (262) GOBP POSITIVE REGULATION OF CHOLESTEROL ESTERIFICATION; (263) GOBP NEGATIVE REGULATION OF CYTOSKELETON ORGANIZATION; (264) GOBP MEMBRANE DOCKING; (265) GOBP NERVE GROWTH FACTOR SIGNALING PATHWAY; (266) GOBP REGULATION OF CHOLESTEROL ESTERIFICATION; (267) GOBP CELL DIFFERENTIATION IN HINDBRAIN; (268) GOBP RRNA CATABOLIC PROCESS; (269) GOBP NEGATIVE REGULATION OF MRNA SPLICING VIA SPLICEOSOME; (270) GOBP CEREBELLAR CORTEX MORPHOGENESIS; (271) GOBP NEGATIVE REGULATION OF MICROTUBULE POLYMERIZATION OR DEPOLYMERIZATION; (272) GOBP POSITIVE REGULATION OF PROTEIN POLYMERIZATION; (273) GOBP METENCEPHALON DEVELOPMENT; (274) GOBP DNA BIOSYNTHETIC PROCESS; (275) GOBP REGULATION OF TRANSCRIPTION INVOLVED IN G1 S TRANSITION OF MITOTIC CELL CYCLE; (276) GOBP MACROPHAGE COLONY STIMULATING FACTOR SIGNALING PATHWAY; (277) GOBP INSULIN SECRETION; (278) GOBP REGULATION OF TRANS SYNAPTIC SIGNALING; (279) GOBP POSTSYNAPSE ASSEMBLY; (280) GOBP NORADRENERGIC NEURON DIFFERENTIATION; (281) GOBP RESPONSE TO POTASSIUM ION; (282) GOBP DEOXYRIBONUCLEOTIDE METABOLIC PROCESS; (283) GOBP AORTA SMOOTH MUSCLE TISSUE MORPHOGENESIS; (284) GOBP CEREBELLAR CORTEX FORMATION; (285) GOBP OOCYTE DIFFERENTIATION; (286) GOCC REPLICATION FORK; (287) GOCC CONDENSED CHROMOSOME; (288) GOCC KINETOCHORE; (289) GOCC CHROMOSOME CENTROMERIC REGION; (290) GOCC NUCLEAR CHROMOSOME; (291) GOCC MIDBODY; (292) GOCC CHROMOSOMAL REGION; (293) GOCC SPINDLE; (294) GOCC MICROTUBULE; (295) GOCC NUCLEAR PROTEIN CONTAINING COMPLEX; (296) GOCC MICROTUBULE CYTOSKELETON; (297) GOCC POLYMERIC CYTOSKELETAL FIBER; (298) GOCC CHROMOSOME; (299) GOCC AXON; (300) GOCC INTRACELLULAR PROTEIN CONTAINING COMPLEX; (301) GOCC SUPRAMOLECULAR COMPLEX; (302) GOCC CATALYTIC COMPLEX; (303) GOCC MICROTUBULE ORGANIZING CENTER; (304) GOCC NUCLEAR BODY; (305) GOCC CONDENSED CHROMOSOME CENTROMERIC REGION; (306) GOCC SPINDLE POLE; (307) GOCC MITOTIC SPINDLE; (308) GOCC SUPRAMOLECULAR POLYMER; (309) GOCC CONDENSED NUCLEAR CHROMOSOME; (310) GOCC LAMELLIPODIUM; (311) GOCC DISTAL AXON; (312) GOCC CENTROSOME; (313) GOCC NEURON PROJECTION; (314) GOCC TERMINAL BOUTON; (315) GOCC GLUTAMATERGIC SYNAPSE; (316) GOCC PEPTIDASE COMPLEX; (317) GOCC CENTRIOLE; (318) GOCC TRANSCRIPTION REPRESSOR COMPLEX; (319) GOCC TRIGLYCERIDE RICH PLASMA LIPOPROTEIN PARTICLE; (320) GOCC MICROTUBULE ASSOCIATED COMPLEX; (321) GOCC SPINDLE MICROTUBULE; (322) GOCC SWI SNF SUPERFAMILY TYPE COMPLEX; (323) GOCC CHROMOSOME TELOMERIC REGION; (324) GOCC SPLICEOSOMAL COMPLEX; (325) GOCC SYNAPTONEMAL STRUCTURE; (326) GOCC CHROMATIN; (327) GOCC CELL BODY; (328) GOCC SAGA COMPLEX; (329) GOCC SITE OF DOUBLE STRAND BREAK; (330) GOCC PRESYNAPSE; (331) GOCC TRANSFERASE COMPLEX; (332) GOCC SCHAFFER COLLATERAL CA1 SYNAPSE; (333) GOCC PROTEIN ACETYLTRANSFERASE COMPLEX; (334) GOCC EXTRINSIC COMPONENT OF CYTOPLASMIC SIDE OF PLASMA MEMBRANE; (335) GOCC ATPASE COMPLEX; (336) GOCC L TYPE VOLTAGE GATED CALCIUM CHANNEL COMPLEX; (337) GOCC SPINDLE MIDZONE; (338) GOCC HISTONE DEACETYLASE COMPLEX; (339) GOCC SITE OF DNA DAMAGE; (340) GOCC NUCLEAR PERIPHERY; (341) GOCC SYNAPSE; (342) GOMF MICROTUBULE BINDING; (343) GOMF TUBULIN BINDING; (344) GOMF CHROMATIN BINDING; (345) GOMF CYTOSKELETAL PROTEIN BINDING; (346) GOMF ADENYL NUCLEOTIDE BINDING; (347) GOMF RIBONUCLEOTIDE BINDING; (348) GOMF TRANSFERASE ACTIVITY TRANSFERRING ONE CARBON GROUPS; (349) GOMF HISTONE BINDING; (350) GOMF HYDROPEROXY ICOSATETRAENOATE DEHYDRATASE ACTIVITY; (351) GOMF VOLTAGE GATED CALCIUM CHANNEL ACTIVITY; (352) GOMF CATALYTIC ACTIVITY ACTING ON DNA; (353) GOMF SEQUENCE SPECIFIC DNA BINDING; (354) GOMF SINGLE STRANDED DNA BINDING; (355) GOMF HYDROLASE ACTIVITY ACTING ON ACID ANHYDRIDES; (356) GOMF SEQUENCE SPECIFIC MRNA BINDING; (357) GOMF DNA REPLICATION ORIGIN BINDING; (358) GOMF STRUCTURAL CONSTITUENT OF CYTOSKELETON; (359) GOMF SINGLE STRANDED DNA HELICASE ACTIVITY; (360) GOMF TAU PROTEIN BINDING; (361) GOMF 3 5 DNA HELICASE ACTIVITY; (362) GOMF CALCIUM ION TRANSMEMBRANE TRANSPORTER ACTIVITY; (363) GOMF DNA DEPENDENT ATPASE ACTIVITY; (364) GOMF GLUTAMATE RECEPTOR ACTIVITY; (365) GOMF RNA BINDING |
| mRNA | Neg. | (1) REACTOME SODIUM PROTON EXCHANGERS; (2) REACTOME METAL SEQUESTRATION BY ANTIMICROBIAL PROTEINS; (3) REACTOME RESPONSE OF EIF2AK1 HRI TO HEME DEFICIENCY; (4) REACTOME RECYCLING OF BILE ACIDS AND SALTS; (5) GOBP POSITIVE REGULATION OF COMPLEMENT ACTIVATION; (6) GOBP POSITIVE REGULATION OF ALKALINE PHOSPHATASE ACTIVITY; (7) GOBP REGULATION OF BILE ACID SECRETION; (8) GOBP NEGATIVE REGULATION OF CARDIAC MUSCLE ADAPTATION; (9) GOBP NEGATIVE REGULATION OF MAST CELL ACTIVATION INVOLVED IN IMMUNE RESPONSE; (10) GOBP NATURAL KILLER CELL MEDIATED IMMUNE RESPONSE TO TUMOR CELL; (11) GOBP FLAVONOID GLUCURONIDATION; (12) GOBP XENOBIOTIC TRANSPORT ACROSS BLOOD BRAIN BARRIER; (13) GOBP NEGATIVE REGULATION OF LEUKOCYTE DEGRANULATION; (14) GOBP REGULATION OF T HELPER 1 CELL DIFFERENTIATION; (15) GOBP ISOPRENOID CATABOLIC PROCESS; (16) GOBP FLAVONOID METABOLIC PROCESS; (17) GOBP NEGATIVE REGULATION OF MAST CELL ACTIVATION; (18) GOBP METANEPHRIC NEPHRON TUBULE EPITHELIAL CELL DIFFERENTIATION; (19) GOBP VESICLE FUSION WITH GOLGI APPARATUS; (20) GOBP NEGATIVE REGULATION OF MYELOID LEUKOCYTE MEDIATED IMMUNITY; (21) GOBP NEGATIVE REGULATION OF PHOSPHOLIPASE ACTIVITY; (22) GOBP ANTIMICROBIAL PEPTIDE PRODUCTION; (23) GOBP NUCLEOTIDE BINDING OLIGOMERIZATION DOMAIN CONTAINING 1 SIGNALING PATHWAY; (24) GOBP NEGATIVE REGULATION OF MAST CELL DEGRANULATION; (25) GOCC GOLGI TRANS CISTERNA; (26) GOMF PROTEIN GLUTAMINE GAMMA GLUTAMYLTRANSFERASE ACTIVITY; (27) GOMF POTASSIUM ION ANTIPORTER ACTIVITY |
| Prot. | Pos. | (1) REACTOME METABOLISM OF POLYAMINES; (2) GOBP NEGATIVE REGULATION OF ACUTE INFLAMMATORY RESPONSE TO ANTIGENIC STIMULUS; (3) GOBP POSITIVE REGULATION OF NEURON APOPTOTIC PROCESS; (4) GOBP POSITIVE REGULATION OF PEROXISOME PROLIFERATOR ACTIVATED RECEPTOR SIGNALING PATHWAY; (5) GOBP REGULATION OF PEROXISOME PROLIFERATOR ACTIVATED RECEPTOR SIGNALING PATHWAY; (6) GOBP NEGATIVE REGULATION OF INFLAMMATORY RESPONSE TO ANTIGENIC STIMULUS; (7) GOBP POSITIVE REGULATION OF NEURON DEATH; (8) GOBP REGULATION OF DENDRITIC SPINE MAINTENANCE; (9) GOBP NEGATIVE REGULATION OF MYOBLAST PROLIFERATION; (10) GOBP REGULATION OF MYELOID LEUKOCYTE MEDIATED IMMUNITY; (11) GOBP REGULATION OF HYPERSENSITIVITY; (12) GOBP TRANSFORMING GROWTH FACTOR BETA1 PRODUCTION; (13) GOBP POSITIVE REGULATION OF OSTEOCLAST DEVELOPMENT; (14) GOBP NEGATIVE REGULATION OF TRANSFORMING GROWTH FACTOR BETA PRODUCTION; (15) GOBP MAINTENANCE OF GASTROINTESTINAL EPITHELIUM; (16) GOBP REGULATION OF HUMORAL IMMUNE RESPONSE MEDIATED BY CIRCULATING IMMUNOGLOBULIN; (17) GOBP NEGATIVE REGULATION OF B CELL PROLIFERATION; (18) GOBP CELLULAR DEFENSE RESPONSE; (19) GOBP CELLULAR RESPONSE TO MINERALOCORTICOID STIMULUS; (20) GOBP REGULATION OF OSTEOCLAST DEVELOPMENT; (21) GOBP RESPONSE TO ELECTRICAL STIMULUS; (22) GOBP GLYOXYLATE METABOLIC PROCESS; (23) GOBP NEGATIVE REGULATION OF TRANSFORMING GROWTH FACTOR BETA1 PRODUCTION; (24) GOBP EPITHELIAL STRUCTURE MAINTENANCE; (25) GOBP CELLULAR RESPONSE TO ALDEHYDE; (26) GOBP SOFT PALATE DEVELOPMENT; (27) GOBP NEGATIVE REGULATION OF HUMORAL IMMUNE RESPONSE MEDIATED BY CIRCULATING IMMUNOGLOBULIN; (28) GOBP OSTEOCLAST DEVELOPMENT; (29) GOBP CORNEA DEVELOPMENT IN CAMERA TYPE EYE; (30) GOCC SODIUM CHANNEL COMPLEX; (31) GOCC ACTIN FILAMENT BUNDLE; (32) GOCC CONTRACTILE FIBER; (33) GOMF UBIQUITIN PROTEIN TRANSFERASE REGULATOR ACTIVITY; (34) GOMF IMMUNOGLOBULIN RECEPTOR ACTIVITY |
| Prot. | Neg. | (1) KEGG SULFUR METABOLISM; (2) REACTOME MHC CLASS II ANTIGEN PRESENTATION; (3) REACTOME TRANSPORT OF FATTY ACIDS; (4) REACTOME CYTOSOLIC SULFONATION OF SMALL MOLECULES; (5) REACTOME COSTIMULATION BY THE CD28 FAMILY; (6) REACTOME TCR SIGNALING; (7) REACTOME MITOCHONDRIAL FATTY ACID BETA OXIDATION OF UNSATURATED FATTY ACIDS; (8) REACTOME ADRENOCEPTORS; (9) REACTOME PD 1 SIGNALING; (10) REACTOME BETA OXIDATION OF LAUROYL COA TO DECANOYL COA COA; (11) GOBP NEGATIVE REGULATION OF GUANYL NUCLEOTIDE EXCHANGE FACTOR ACTIVITY; (12) GOBP REGULATION OF GUANYL NUCLEOTIDE EXCHANGE FACTOR ACTIVITY; (13) GOBP LABYRINTHINE LAYER MORPHOGENESIS; (14) GOBP AMINO SUGAR METABOLIC PROCESS; (15) GOBP REGULATION OF GLUTAMATE RECEPTOR SIGNALING PATHWAY; (16) GOBP EMBRYONIC PLACENTA MORPHOGENESIS; (17) GOBP 3 PHOSPHOADENOSINE 5 PHOSPHOSULFATE METABOLIC PROCESS; (18) GOBP REGULATION OF DEFENSE RESPONSE TO BACTERIUM; (19) GOBP LYMPHOCYTE CHEMOTAXIS; (20) GOBP RECEPTOR TRANSACTIVATION; (21) GOBP REGULATION OF ESTABLISHMENT OR MAINTENANCE OF CELL POLARITY; (22) GOBP NEGATIVE REGULATION OF T CELL MIGRATION; (23) GOBP NEGATIVE REGULATION OF LYMPHOCYTE MIGRATION; (24) GOBP POSITIVE REGULATION OF I KAPPAB PHOSPHORYLATION; (25) GOBP LONG CHAIN FATTY ACID CATABOLIC PROCESS; (26) GOBP EXPLORATION BEHAVIOR; (27) GOBP EPINEPHRINE TRANSPORT; (28) GOBP RESPONSE TO CHEMOKINE; (29) GOBP NEGATIVE REGULATION OF CELL PROLIFERATION INVOLVED IN KIDNEY DEVELOPMENT; (30) GOBP REGULATION OF EPINEPHRINE SECRETION; (31) GOBP HISTONE H3 K14 ACETYLATION; (32) GOBP TOLL LIKE RECEPTOR 7 SIGNALING PATHWAY; (33) GOBP NEGATIVE REGULATION OF NOREPINEPHRINE SECRETION; (34) GOCC INTRINSIC COMPONENT OF ENDOPLASMIC RETICULUM MEMBRANE; (35) GOCC MHC PROTEIN COMPLEX; (36) GOCC GOLGI APPARATUS SUBCOMPARTMENT; (37) GOCC INTRINSIC COMPONENT OF GOLGI MEMBRANE; (38) GOCC ORGANELLE SUBCOMPARTMENT; (39) GOCC CLATHRIN COATED VESICLE MEMBRANE; (40) GOCC ER TO GOLGI TRANSPORT VESICLE MEMBRANE; (41) GOCC COATED VESICLE MEMBRANE; (42) GOCC NUCLEAR OUTER MEMBRANE ENDOPLASMIC RETICULUM MEMBRANE NETWORK; (43) GOCC ENDOSOME; (44) GOCC CLATHRIN COATED ENDOCYTIC VESICLE; (45) GOCC COPII COATED ER TO GOLGI TRANSPORT VESICLE; (46) GOCC MHC CLASS II PROTEIN COMPLEX; (47) GOMF HYDROLASE ACTIVITY ACTING ON CARBON NITROGEN BUT NOT PEPTIDE BONDS IN CYCLIC AMIDES; (48) GOMF CCR2 CHEMOKINE RECEPTOR BINDING; (49) GOMF ALPHA ADRENERGIC RECEPTOR ACTIVITY; (50) GOMF ADRENERGIC RECEPTOR ACTIVITY; (51) GOMF OXIDOREDUCTASE ACTIVITY ACTING ON THE CH CH GROUP OF DONORS |

| **Table S13.** Gene-set enrichment analysis (GSEA) results of phosphoproteomics that were associated with rs75776403. | | | | | | | |
| --- | --- | --- | --- | --- | --- | --- | --- |
| **No.** | **Pathway** | **Source** | **P Value** | **Padj.** | **log2err** | **ES** | **NES** |
| 1 | PATH-NP ANDROGEN RECEPTOR PATHWAY | gluC | 0.03235 | 0.24211 | 0.32178 | 0.95697 | 1.37629 |
| 2 | PATH-NP CCR7 PATHWAY | gluC | 0.00803 | 0.10973 | 0.38073 | 0.9723 | 1.46405 |
|  |  | max. | 0.04208 | 0.66684 | 0.32178 | 0.84201 | 1.43285 |
| 3 | PATH-NP EGFR1 PATHWAY | trypsin | 0.0203 | 0.85854 | 0.35249 | 0.49086 | 1.34024 |
| 4 | PATH-NP GASTRIN PATHWAY | max. | 0.02627 | 0.66684 | 0.35249 | 0.90314 | 1.43852 |
| 5 | PATH-NP GLP1 PATHWAY | gluC | 0.00365 | 0.10973 | 0.43171 | 0.92777 | 1.58817 |
| 6 | PATH-NP IL11 PATHWAY | gluC | 0.03543 | 0.24211 | 0.32178 | 0.95465 | 1.37296 |
| 7 | PATH-NP TSLP PATHWAY | gluC | 0.00714 | 0.10973 | 0.40702 | 0.72505 | 1.58858 |
| 8 | PATH-WP PI3K-Akt Signaling Pathway | gluC | 0.01082 | 0.11094 | 0.38073 | 0.80494 | 1.59418 |
|  |  | max. | 0.04088 | 0.66684 | 0.28201 | 0.65813 | 1.39251 |

**Fig. S2.** Pairwise correlation plot showing a gene-gene correlation between genes that are involved in the cellular response to thyroid hormone stimulus (purple) and corticosteroid receptor signaling pathways (orange). *CLEC18A* gene is marked using a red dot.

| **Table S14.** Association between (A) rs75776403 or (B) *CLEC18A* mRNA level with thyroid hormone axial genes. | | | | | | |
| --- | --- | --- | --- | --- | --- | --- |
|  | **(A) rs75776403** | |  | **(B) *CLEC18A* mRNA** | | |
| **Gene** | ***β*** | ***P*** |  | ***ρ*** | **r^2^** | ***P*** |
| *TG* | -0.14 | **0.0307** |  | 0.12 | 0.01 | **2.20×10^-4^** |
| *THRB* | -0.22 | **0.0436** |  | 0.12 | 0.01 | **2.90×10^-4^** |
| *TSHB* | -0.01 | 0.2770 |  | 0.04 | 0.00 | 0.2237 |
| *TRH* | 0.06 | 0.3068 |  | 0.06 | 0.00 | 0.0540 |
| *TSHR* | 0.06 | 0.4606 |  | 0.13 | 0.02 | **3.43×10^-5^** |
| *THRA* | 0.06 | 0.5456 |  | 0.11 | 0.01 | **1.09×10^-3^** |
| *TRHR* | 0.00 | 0.9128 |  | 0.10 | 0.01 | **2.39×10^-3^** |
| A *P* value of <0.05 was highlighted in **bold**. | | | | | | |

| **Table S15.** Gene set of cellular response to thyroid hormone stimulus and corticosteroid receptor signaling pathway in gene ontology (GO) biological process (BP) database. | | | |
| --- | --- | --- | --- |
| **GO term** | **No.** | **Cellular response to thyroid hormone stimulus**  **(GO ID:_0097067)** | **Corticosteroid receptor signaling pathway**  **(GO ID:_0031958)** |
| Gene set | 1 | *BRD8* | *ARID1A* |
|  | 2 | *CTSB* | *ARNTL* |
|  | 3 | *CTSH* | *CALR* |
|  | 4 | *CTSL* | *CLOCK* |
|  | 5 | *CTSS* | *CRY1* |
|  | 6 | *GAS2L1* | *CRY2* |
|  | 7 | *GATA1* | *JAK2* |
|  | 8 | *GCLM* | *LMO3* |
|  | 9 | *GHSR* | *NEDD4* |
|  | 10 | *KIT* | *NR3C1* |
|  | 11 | *KLF9* | *PER1* |
|  | 12 | *LMO2* | *PHB* |
|  | 13 | *MED1* | *PPP5C* |
|  | 14 | *PPARGC1A* | *YWHAH* |
|  | 15 | *RDX* | *-* |
|  | 16 | *THRB* | *-* |

| **Table S16.** Association between rs75776403 and corticosteroid receptor signaling or thyroid-stimulated pathway genes. | | | | | |
| --- | --- | --- | --- | --- | --- |
|  | **mRNA** | |  | **Protein** | |
|  | ***β*** | ***P*** |  | ***β*** | ***P*** |
| *ARID1A* | 0.24 | **0.0015** |  | 0.19 | 0.1133 |
| *YWHAH* | 0.15 | **0.0170** |  | 0.12 | 0.2411 |
| *CRY1* | 0.18 | **0.0359** |  | -0.33 | 0.2359 |
| *LMO3* | 0.31 | **0.0385** |  | 4.69 | **0.0261** |
| *CLOCK* | 0.08 | 0.2327 |  | -0.03 | 0.8367 |
| *ARNTL* | 0.11 | 0.2734 |  | 0.13 | 0.5985 |
| *CRY2* | 0.05 | 0.4273 |  | - | - |
| *NEDD4* | -0.07 | 0.4761 |  | 0.04 | 0.7447 |
| *CALR* | 0.05 | 0.5252 |  | -0.05 | 0.6034 |
| *NR3C1* | -0.07 | 0.5390 |  | 0.06 | 0.7287 |
| *JAK2* | 0.04 | 0.5640 |  | 0.62 | **0.0340** |
| *PPP5C* | 0.03 | 0.6255 |  | -0.08 | 0.4492 |
| *PER1* | 0.02 | 0.7771 |  | 0.04 | 0.8474 |
| *PHB* | -0.01 | 0.9223 |  | -0.10 | 0.2594 |
| *THRB* | -0.22 | **0.0436** |  | - | **-** |
| *CTSH* | -0.27 | 0.1943 |  | -0.38 | 0.1478 |
| *LMO2* | -0.14 | 0.3341 |  | -2.14 | 0.3624 |
| *GATA1* | -0.08 | 0.3802 |  | -0.63 | 0.4448 |
| *MED1* | 0.05 | 0.3944 |  | -0.04 | 0.5055 |
| *GHSR* | -0.01 | 0.4252 |  | - | - |
| *CTSL* | -0.12 | 0.5518 |  | 0.01 | 0.9402 |
| *KIT* | 0.09 | 0.5721 |  | -0.19 | 0.6749 |
| *CTSS* | 0.10 | 0.5906 |  | -0.39 | 0.2111 |
| *GAS2L1* | 0.04 | 0.6853 |  | 0.14 | 0.4245 |
| *KLF9* | -0.03 | 0.7811 |  | - | - |
| *GCLM* | 0.02 | 0.8441 |  | 0.13 | 0.3753 |
| *CTSB* | 0.01 | 0.9468 |  | 0.05 | 0.7739 |
| *PPARGC1A* | -0.01 | 0.9576 |  | - | - |
| *RDX* | 0.00 | 0.9728 |  | 0.05 | 0.7426 |
| *BRD8* | 0.00 | 0.9816 |  | 0.03 | 0.7441 |
| Genes implicated in corticosteroid receptor signaling were labeled in grey color. Genes implicated in thyroid-stimulated pathway were labeled in white color. A *P* value of <0.05 was highlighted in **bold**. Protein expression of genes *CRY2*, *THRB*, *GHSR*, *KLF9*, and *PPARGC1A* were missing. | | | | | |

| **Table S17.** Association between *CLEC18A* and thyroid stimulated pathways or corticosteroid receptor signaling genes in adrenal gland, liver, ovary, and testis tissues from GTEx portal. | | | | | | | | | | | | | | | |
| --- | --- | --- | --- | --- | --- | --- | --- | --- | --- | --- | --- | --- | --- | --- | --- |
|  | **Adrenal gland (*N*=275)** | | |  | **Liver (*N*=251)** | | |  | **Ovary (*N*=195)** | | |  | **Testis (*N*=406)** | | |
| **mRNA** | ***ρ*** | ***r*^2^** | ***P*** |  | ***ρ*** | ***r*^2^** | ***P*** |  | ***ρ*** | ***r*^2^** | ***P*** |  | ***ρ*** | ***r*^2^** | ***P*** |
| *BRD8* | 0.20 | 0.04 | **1.12×10^-3^** |  | 0.35 | 0.12 | **4.81×10^-8^** |  | 0.21 | 0.04 | **4.76×10^-3^** |  | -0.08 | 0.01 | 0.1212 |
| *MED1* | -0.12 | 0.01 | 0.0649 |  | 0.19 | 0.04 | **3.43×10^-3^** |  | -0.11 | 0.01 | 0.1422 |  | 0.00 | 0.00 | 0.9911 |
| *KLF9* | 0.03 | 0.00 | 0.6077 |  | -0.05 | 0.00 | 0.4492 |  | -0.16 | 0.03 | **0.0276** |  | -0.04 | 0.00 | 0.4752 |
| *CTSL* | -0.18 | 0.03 | **3.24×10^-3^** |  | 0.02 | 0.00 | 0.7361 |  | -0.18 | 0.03 | **0.0174** |  | -0.04 | 0.00 | 0.4684 |
| *CTSB* | -0.11 | 0.01 | 0.0862 |  | 0.12 | 0.01 | 0.0766 |  | -0.04 | 0.00 | 0.5812 |  | 0.05 | 0.00 | 0.3364 |
| *GAS2L1* | 0.20 | 0.04 | **1.09×10^-3^** |  | 0.20 | 0.04 | **3.08×10^-3^** |  | 0.22 | 0.05 | **3.27×10^-3^** |  | 0.26 | 0.07 | **4.06×10^-7^** |
| *KIT* | 0.22 | 0.05 | **3.70×10^-4^** |  | 0.00 | 0.00 | 0.9763 |  | 0.00 | 0.00 | 0.9713 |  | -0.03 | 0.00 | 0.5538 |
| *PPARGC1A* | 0.02 | 0.00 | 0.6968 |  | 0.03 | 0.00 | 0.6656 |  | 0.12 | 0.01 | 0.1087 |  | 0.02 | 0.00 | 0.7519 |
| *GCLM* | -0.26 | 0.07 | **2.24×10^-5^** |  | 0.04 | 0.00 | 0.5694 |  | -0.12 | 0.01 | 0.1135 |  | -0.05 | 0.00 | 0.3709 |
| *RDX* | -0.05 | 0.00 | 0.4640 |  | 0.13 | 0.02 | 0.0509 |  | 0.03 | 0.00 | 0.6749 |  | 0.11 | 0.01 | **0.0293** |
| *GATA1* | 0.03 | 0.00 | 0.5833 |  | 0.02 | 0.00 | 0.7672 |  | 0.22 | 0.05 | **2.88×10^-3^** |  | -0.10 | 0.01 | 0.0666 |
| *CTSS* | -0.02 | 0.00 | 0.7648 |  | 0.11 | 0.01 | 0.1143 |  | 0.04 | 0.00 | 0.6212 |  | 0.04 | 0.00 | 0.4882 |
| *GHSR* | 0.01 | 0.00 | 0.8327 |  | 0.01 | 0.00 | 0.8699 |  | -0.11 | 0.01 | 0.1571 |  | 0.05 | 0.00 | 0.3683 |
| *LMO2* | 0.21 | 0.04 | **6.91×10^-4^** |  | 0.12 | 0.01 | 0.0836 |  | 0.21 | 0.04 | **5.21×10^-3^** |  | 0.06 | 0.00 | 0.2359 |
| *CTSH* | 0.07 | 0.00 | 0.2613 |  | 0.16 | 0.03 | **0.0146** |  | -0.02 | 0.00 | 0.7623 |  | -0.04 | 0.00 | 0.5037 |
| *THRB* | -0.02 | 0.00 | 0.8076 |  | -0.02 | 0.00 | 0.8076 |  | 0.09 | 0.01 | 0.2250 |  | -0.01 | 0.00 | 0.8309 |
| *ARID1A* | 0.00 | 0.00 | 0.9924 |  | 0.26 | 0.07 | **9.00×10^-5^** |  | -0.07 | 0.01 | 0.3443 |  | 0.07 | 0.01 | 0.1647 |
| *JAK2* | 0.09 | 0.01 | 0.1496 |  | 0.08 | 0.01 | 0.2493 |  | 0.08 | 0.01 | 0.2699 |  | -0.08 | 0.01 | 0.1214 |
| *CRY1* | -0.07 | 0.00 | 0.2733 |  | 0.09 | 0.01 | 0.1953 |  | -0.06 | 0.00 | 0.3899 |  | -0.06 | 0.00 | 0.2797 |
| *CLOCK* | 0.04 | 0.00 | 0.5556 |  | 0.19 | 0.03 | **5.23×10^-3^** |  | -0.08 | 0.01 | 0.2671 |  | -0.01 | 0.00 | 0.7930 |
| *NR3C1* | 0.04 | 0.00 | 0.4945 |  | 0.20 | 0.04 | **2.40×10^-3^** |  | 0.13 | 0.02 | 0.0913 |  | 0.04 | 0.00 | 0.4871 |
| *PPP5C* | -0.05 | 0.00 | 0.4311 |  | 0.06 | 0.00 | 0.3946 |  | -0.01 | 0.00 | 0.8555 |  | -0.01 | 0.00 | 0.8021 |
| *PER1* | 0.24 | 0.06 | **9.26×10^-5^** |  | 0.06 | 0.00 | 0.3443 |  | -0.05 | 0.00 | 0.5011 |  | -0.05 | 0.00 | 0.3391 |
| *CALR* | -0.21 | 0.04 | **7.50×10^-4^** |  | 0.07 | 0.00 | 0.3241 |  | -0.11 | 0.01 | 0.1452 |  | 0.01 | 0.00 | 0.8529 |
| *CRY2* | 0.11 | 0.01 | 0.0855 |  | 0.09 | 0.01 | 0.2007 |  | 0.07 | 0.00 | 0.3853 |  | 0.18 | 0.03 | **4.18×10^-4^** |
| *ARNTL* | 0.01 | 0.00 | 0.8991 |  | 0.15 | 0.02 | **0.0203** |  | -0.07 | 0.01 | 0.3290 |  | 0.20 | 0.04 | **1.76×10^-4^** |
| *PHB* | -0.17 | 0.03 | **7.48×10^-3^** |  | 0.06 | 0.00 | 0.3709 |  | -0.13 | 0.02 | 0.0891 |  | 0.15 | 0.02 | **4.20×10^-3^** |
| *YWHAH* | -0.04 | 0.00 | 0.4742 |  | 0.03 | 0.00 | 0.6850 |  | 0.01 | 0.00 | 0.8459 |  | 0.08 | 0.01 | 0.1509 |
| *NEDD4* | -0.09 | 0.01 | 0.1447 |  | 0.14 | 0.02 | **0.0362** |  | 0.23 | 0.05 | **1.73×10^-3^** |  | -0.03 | 0.00 | 0.5465 |
| *LMO3* | 0.11 | 0.01 | 0.0910 |  | 0.19 | 0.04 | **3.74×10^-3^** |  | 0.16 | 0.02 | **0.0373** |  | -0.10 | 0.01 | 0.0691 |
| *N*, sample size. Genes implicated in thyroid stimulated pathways were labeled in white. Genes implicated in corticosteroid receptor signaling were labeled in grey. *P* Value <0.05 were highlighted in **bold**. | | | | | | | | | | | | | | | |
